# Pollution and health risk assessment of drinking water sources within artisanal and small-scale gold mining areas: a case of Asankrangwa District in Ghana

**DOI:** 10.1007/s11356-026-37463-y

**Published:** 2026-02-10

**Authors:** Eric Danso-Boateng, Ebenezer Adom, Prince Appiah Owusu, Roland Songotu Kabange

**Affiliations:** 1 School of Chemical and Process Engineering, University of Leeds, Leeds, LS2 9JT UK; 2 Department of Chemical Engineering, Kwame Nkrumah University of Science and Technology, Private Mail Bag, University Post Office, Kumasi, Ghana; 3 Department of Civil Engineering, Kumasi Technical University, P.O. Box 854, Kumasi, Ghana

**Keywords:** Cancer health risk, Cyanide contamination, Gold mining pollution, Heavy metal contamination, Heavy metal pollution index, Mercury contamination, Water quality index

## Abstract

Artisanal and small-scale gold mining (ASGM) is common in many developing countries; while it contributes to their socio-economic development, it also causes environmental pollution. This study examined the impact of ASGM activities on the quality of drinking water sources in the Asankrangwa District in Ghana, in terms of water quality index, heavy metal pollution index models, and human health risks. The results of physicochemical parameters indicate that 40.0% of all water sources are unsuitable for consumption. Heavy metal pollution indices indicated that 45.0% of the water sources were extremely polluted, and 25.0% were moderately polluted. For instance, cadmium (Cd) concentrations across all water sources reached levels up to 23 times the WHO limit (3.0 × 10^–3^ mg/L). Additionally, mercury (Hg) concentrations in 75.0% of the water sources (2.0 × 10^–3^–7.2 × 10^–1^ mg/L) exceed the maximum contaminant limit (2.0 × 10^–3^ mg/L) by approximately 361 times. Streams, in particular, exhibit higher Hg levels than groundwaters. Analysis revealed that 90% of the water sources pose a significant carcinogenic health risk upon consumption, with lifetime cancer risk values exceeding 1.0 × 10^−4^. The findings revealed that the pollution level is reduced and water quality improved 5 km from the ASGM sites; however, the water sources require treatment to render them suitable for consumption.

**Supplementary Information:**

The online version contains supplementary material available at 10.1007/s11356-026-37463-y.

## Introduction

Artisanal and small-scale gold mining (ASGM) and quarrying operations are popular in sub-Saharan Africa, South America, and Southeast Asia, and the degree of proliferation varies in different countries (Diaz et al. 2020; Hilson and McQuilken 2014; Lahiri-Dutt 2008). ASGM provides substantial socio-economic benefits to these countries, particularly for local communities. According to estimates, nearly 13 million people in about thirty countries work in the ASGM sector. Around 80 to 100 million people depend on this sector for their livelihood (Andrews 2015; Hilson and Garforth 2012; Tschakert 2009). In Ghana, ASGM (including illegal mining operations referred to as ‘galamsey’) contributed 31% of the total gold production in (Apau, et al., 2016). It directly employs over one million people and creates opportunities for more than five million people in the downstream industries and markets (Vande Pallen 2017). It has been estimated that ASGM contributes 2.0% to Ghana’s gross domestic product (Gatune and Besada 2020).

Despite the multiple benefits received from the sector, the repercussions of ASGM have been disastrous in recent years, with severe negative environmental, social, and economic consequences. For example, ASGM is a driver of environmental degradation, including deforestation, land degradation, agricultural losses, soil erosion, and water pollution (Aryee 2012; Darkwah 2017; Mensah 2018), thereby endangering aquatic ecosystems. Water pollution stems from inefficient ore processing, tailing facilities, and waste dump sites. The level of contamination fluctuates and is dependent on the size of the mine and the processing procedures used (Aryee et al. 2003). According to the International Growth Centre (IGC), it will cost Ghana about USD 250 million to recover lands and water sources destroyed by ASGM and/or galamsey operations (Dzansi and Telli 2019). It is envisioned that, in the next 8 to 10 years, Ghana may have to import water if drastic measures are not taken (Quarshie 2017). Despite this, there has been a lack of specific details regarding how ASGM activities affect the drinking water quality of localities in Ghana.

Exploration, mining, and processing are all involved in obtaining minerals. In ASGM, minerals are primarily recovered from alluvial deposits near waterways. Mercury (Hg) is used to create an alloy amalgam of gold, which is then burned to vapourise the Hg, leaving pure gold (Hayford et al. 2008). The ASGM industry uses approximately 200 metric tons of Hg annually, accounting for over 30.0% of the industrial applications of Hg (Gallo Corredor et al. 2021; Yoshimura et al. 2021). Due to the elementary techniques used in ASGM, the Hg used in the cyanidation process, as well as its presence in the tailings, is an issue during gold processing. A large fraction of the Hg is discharged into the environment (Gutiérrez-Mosquera et al. 2021). Sub-Saharan Africa ranks as the third-largest emitter of Hg pollution from ASGM, contributing 8.0% of the global total (Diaz et al. 2020). In Ghana, ASGM operations release an estimated 5.0 tons of Hg annually (Asklund and Eldvall 2005). Mercury is hazardous to one’s health and the environment (Matlock et al. 2002). This is a global concern due to its high potential to form complexes with organic substances present in the environment, which can cause the formation of persistent and harmful noxious compounds such as methylmercury (Casso-Hartmann et al. 2022).

Target 6.1 of the United Nations Sustainable Development Goal (SDG) necessitates ‘universal and equitable access to safe and affordable drinking water for all’ (United Nations 2015). ‘Safe’ drinking water, according to international policy, is water free from pathogens and high levels of toxic substances (WHO 2011). Toxic metals such as Hg, lead (Pb), cadmium (Cd), selenium (Se), arsenic (As), and uranium (U) are known to be the cause of large-scale health problems such as kidney disease, cancer, cardiovascular disease, and hypertension (Chowdhury et al. 2018; Houston 2007; Rauh and Margolis 2016; WHO 2011). Environmental Pb exposure alone contributes to about 100,000 deaths and 10 million disability-adjusted life years annually (Lim et al. 2012) and has been linked to irreversible neurodevelopmental impairment, particularly in children and developing foetuses (Rauh and Margolis 2016; Sanders et al. 2009; WHO 2011).

The use of groundwater is extremely important. In terms of contamination, groundwater is more dependable than streams, as it is naturally protected, less influenced by drought (even when close to the point of use), and does not require significant treatment (Cobbina et al. 2010). However, chemical weathering, soil leaching, the decay of plants, and other sources near the ground surface can contaminate groundwater (Amano et al. 2020). These major processes are influenced by geological and geochemical settings, as well as the chemical and biological properties of the pollutants (Nagaraju et al. 2014). This can pollute adjacent populations, particularly those who rely on such bodies of water for drinking and other household reasons.

ASGM causes substantial environmental risks, as well as serious health and safety issues for the mine workers and people in the neighbouring areas. The level of environmental impact is largely determined by the mining methods and processes used (Mensah et al. 2015). The environmental damage caused by ASGM activities and the contamination caused to water bodies is widely acknowledged. For instance, Mantey et al. (2020) monitored Hg concentrations in the soil, surface drainage, sludge, and solid wastes across different ASGM operations in three assemblies in Ghana and found high levels of Hg. Wiafe et al. (2022) reported high levels of Hg and Cd in soil and streams within an ASGM catchment area in Prestea Huni-Valley District in Ghana. Bessah et al. (2021) found high levels of copper (Cu), iron (Fe), As, and Hg in the Pra River Basin near ASGM areas in Southern Ghana above the permissible levels for irrigation. Casso-Hartmann et al. (2022) examined the physicochemical quality, microbiological, and Hg pollution of water bodies within ASGM areas in the Northern region of Cauca in Colombia, revealing that most of the water sources did not meet the quality and safety standards. Obiri et al. (2016) analysed heavy metals in rivers and dregs in the Prestea Huni Valley District of Ghana and found a high hazard quotient (HQ) and cancer health risk (CR) above the guidance limits in most cases. Galarza et al. (2023) found that the total CR for children and adults caused by As and Pd in streams and sediments in all mining sites in the Northeast Andean of the Ecuadorian Amazon exceeded the permissible threshold up to three times.

However, limited research has been done to quantify the degree of water quality and contamination, heavy metal pollution, and the impact of the distance between ASGM activities and water sources on human health risks. Hence, there is still a lack of understanding in these areas, as previous studies on ASGM’s impact on water sources have been generic and focused on streams and sediments. This study, therefore, addresses the following objectives: (i) investigate the level of physicochemical characteristics, cyanide (CN), and heavy metals in drinking water sources within an ASGM area, and determine the contaminants exceeding applicable standards and guidelines; (ii) evaluate water quality index (WQI); (iii) determine heavy metal pollution and evaluation index; (iv) assess non-carcinogenic and carcinogenic health risks of the water sources to understand the health risks associated with ASGM activities; and (v) analyse the effect of distance from the ASGM site on WQI, HPI/HEI and human health risk. This research can serve as a blueprint for policymakers in Ghana and other ASGM operating countries to assess the potential effects of ASGM on water pollution and the connected health risks. It will guide the development of the appropriate interventions to address this menace.

## Materials and methods

### Study area

The study area was Asankrangwa, the capital of the Wassa Amenfi West Municipal Assembly in the Western Region of Ghana. The Western Region is identified as one of Ghana’s hotspots for ASGM operations (Mantey et al. 2016; Owusu-Nimo et al. 2018). Asankrangwa is located in the geologically significant Sefwi-Bibiani Belt, known for its gold deposits. The region also benefits from a hydrologically rich environment, with rivers and water bodies that are vital for various purposes, including agriculture, drinking water supply, and industrial use. The Tano River, for instance, flows through the region, significantly affecting the area’s hydrology. Within the geological setting of Asankrangwa are various geological structures, specifically fault zones, shear zones, and folded rock formations. These structures influence the localisation of mineral deposits, including gold.

### Sampling

**Fig. 1:**
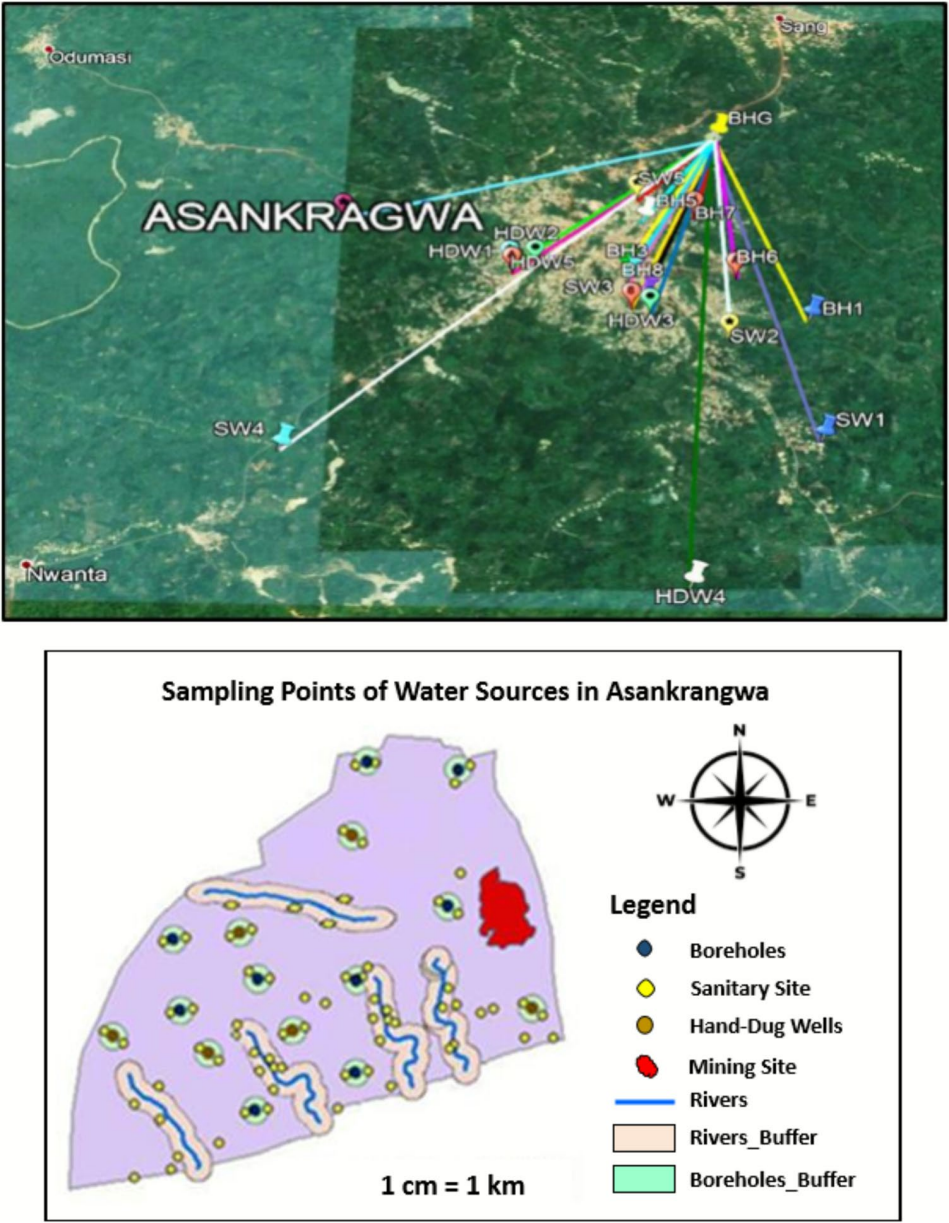
A map showing the sampling points of the water sources in the study

Twenty different water sources in the ASGM area were collected and tested to assess the drinking water quality. Six hand-dug wells (HDW), nine boreholes (BH), and five streams or surface water (SW) samples were taken and analysed. The sampling locations, which were chosen using degrees, minutes, and seconds as latitudes and longitudes, were then mapped using Google Earth Software version 7.3, which provided satellite images of the ASGM study area. The measurements of the sampling points (water sources) to the ASGM site were then accessed using Google Earth for the analysis, as shown in Fig. 1, and the geographical locations are summarised in Table S1 in the Supplementary Materials. The sampling was done between December 2019 and May 2020. However, due to security threats, repeated samples could not be taken during the main rainy season to investigate the effect of weather conditions on water quality and the degree of pollution of the water sources.

The water samples were taken in Teflon vessels washed with tap water and rinsed with deionised water before sampling. To avoid any possible contamination, the bottles were acid-washed with 10.0% (v/v) hydrochloric acid (HCl) following the standard methods provided by the American Public Health Association (APHA 2012). The washed bottles were again rinsed with the water samples at each sampling station. At each visit, blind field water samples were taken, one for heavy metal analysis and the other for physicochemical parameter analysis. The boreholes in the research area have shallow depths between 30.0 and 96.0 m (mean of 44.4 m). Direct sample collection was the primary approach for sampling SW. A feasible and safe access route that was representative of the area was determined to collect SW samples (streams). To ensure proper mixing, the sample container was stirred three times. The sample container was submerged in the streamflow direction and filled to a depth of about 0.3 m beneath the water surface. The groundwater samples were taken straight from the wellheads. The samples were acidified for heavy metal analysis in the field by adding two drops of concentrated nitric acid (HNO_3_), which also helped to preserve the samples as per the standard procedures provided by APHA and the U.S. Geological Survey (USGS) (APHA 2012; USGS 2006). The bottled samples were kept cold in an insulated ice cooler box until they were delivered to the laboratory for analysis.

### Analytical methods

Total dissolved solids (TDS), pH, and electrical conductivity (EC) were analysed in situ during sampling using a multiparameter PH/ISE/EC/DO/Turbidity meter (HI 9829, Hanna Instruments, Leighton Buzzard, UK), according to APHA (2012) standard guidelines. A turbidimeter (HACH 2100 N, HACH, Manchester, UK) was used to measure the turbidity of the water samples. Titrimetric methods were employed to determine the chloride (Cl^−^), alkalinity, total hardness (TH), calcium hardness (Ca^2+^), and magnesium hardness (Mg^2+^) following APHA (2012) standard guidelines.

The concentrations of heavy metals such as Hg, Cu, Pb, Fe, Cd, and manganese (Mn) were analysed according to the APHA (2012) standard techniques using an Atomic Absorption Spectrophotometer (AAS) (NovAA 400 pp, Analytik Jena AG, Jena, Germany). The efficiency of the AAS instrument and the analytical method was validated using parameters such as the instrumental detection limit (IDL), method detection limit (MDL), quantification limit (LOQ), precision, and accuracy. As shown in Table S2 in the Supplementary Materials, all the recovery values were within the acceptable range of 80.0–120.0%. Also, the percentage relative standard deviation (%RSD) values for all the metals in the spiked sample were below 7.7%, below the required control limit of ≤ 10.0%. Following calibration of the AAS instrument, deionised water was used for serial dilution of the intermediate standard solution (50.0 mL of 10.0 mg/L) to prepare 5 series of the working standard solutions. The standard concentrations of the heavy metals and their absorbance were used to plot a five-point calibration graph.

An automated wet chemistry analyser (Skalar San^++^, Skalar Analytical B.V., Breda, The Netherlands) was used to analyse CN concentrations in the water samples. The complex-bound CN is broken down using UV light in a stream that flows continuously at a pH of 3.8 to determine the CN compounds. To block the conversion of thiocyanate into CN, a UV light lamp with a wavelength of 312 nm and a borosilicate glass decomposition spiral was used as a filter. With the use of a spectrophotometer (HACH DR 6000, HACH, Manchester, UK), the anions and cations were identified. Using standard reference materials, the device was calibrated for the quantitative analysis of sulphides, carbonates, and silicates.

Triplicate analyses were performed to ensure reproducibility of the results. Blanks were used to ensure there was no contamination during the sample preparation process.

### Water quality index

The WQI provides an effective way of evaluating the overall quality of drinking water. The classification system by WQI indicates the fitness of water for consumption (Kawo and Karuppannan 2018; Rana et al. 2018). The WQI calculation required assigning a weight (*w*_*i*_) to each water sample and then using this quantity to calculate the relative weight (*W*_*i*_) and the quality rating scale (*q*_*i*_). The parameters with the most impact on drinking water quality were assigned the highest value of 5.0, and those with the least impact were awarded the lowest value of 1.0 (Singh et al. 2016; Srinivasamoorthy et al. 2008; Amano et al. 2020). Alkalinity, pH, TH, TDS, EC, Cl^−^, Ca^2+^, Mg^2+^, CN, and turbidity were all assigned *w*_*i*_ values.

The *W*_*i*_ was calculated using the equation by Rana et al. (2018) as follows:

1 $${W}_{i}=\frac{{W}_{i}}{\sum_{i=1}^{n}{W}_{i}}$$

where *n* is the number of parameters measured. The calculated *W*_*i*_ values are provided in Table S3 in the Supplementary Material.

The quality rating scale of each parameter was calculated by dividing the concentration of each parameter in the water sample (*C*_*i*_ in mg/L) by its corresponding WHO (2011) or Ghana Standards Authority, GSA (2013) standards (*S*_*i*_ in mg/L), and finally multiplying the results by 100 (Rana et al. 2018), as follows:

2 $${q}_{i}= \frac{{C}_{i}}{{S}_{i}} \times 100$$

WQI was then calculated using Eq. (3) (Rana et al. 2018):

3 $$\mathrm{WQI}= \sum\nolimits_{i=1 }^{n}{q}_{i}\times {W}_{i}$$

The water quality rating based on the WQI values is given by Sahu and Sikdar (2008) as follows: excellent water (WQI < 50.0), good water (50.0–100.0), poor water (100.0–200.0), very poor water (200.0–300.0), and water unsuitable for drinking (WQI > 300.0).

### Heavy metal pollution index

HPI is an effective way to characterise and evaluate water quality regarding heavy metal contamination (Reza and Singh 2010). To create the HPI technique, a weightage (*W*_*i*_) was assigned to each parameter by the proportional importance of each quality in question. HPI was calculated according to Eq. (4) (Mohan et al. 1996):

4 $$\mathrm{HPI}=\frac{\sum_{i=1}^{n}{W}_{i}\times {Q}_{i}}{\sum_{i=1}^{n}{W}_{i}}$$

where *Q*_*i*_ is the sub-index of the *i*th parameter, which was calculated by using Eq. (5) as follows:

5 $${Q}_{i}=\sum\nolimits_{i=1}^{n}\frac{\left\{{M}_{i}-{I}_{i}\right\}}{{S}_{i}-{I}_{i}}\times 100$$

where *M*_*i*_ is the heavy metal concentration of the ith parameter (of the water sample), *S*_*i*_ is the highest permissible (standard) value, and *I*_*i*_ is the maximum ideal or desirable (standard) value for drinking water per the WHO (2011) and GSA (2013) standards. Table S4 in the Supplementary Materials shows the HPI calculation of the heavy metals.

### Heavy metal evaluation index

The HEI approach, similar to HPI, determines the total quality of water based on the heavy metals in the water. The HEI was determined according to Eq. (6) (Edet and Offiong 2002):

6 $$\mathrm{HEI}=\sum\nolimits_{i=1}^{n}\frac{{H}_{c}}{{H}_{\mathrm{mac}}}$$

where *H*_*c*_ is the detected value of the ith parameter in the water and *H*_mac_ is the highest desirable standard value (for drinking water) of the *i*th parameter per GSA (2013) or WHO (2011) standards.

### Health risk assessment

The health risk of the water sources was evaluated by quantifying the risk level of the heavy metals and CN, which were characterised by cancer and non-cancer health risks (Sun et al. 2015).

#### Non-carcinogenic health risk analysis

The human non-carcinogenic health effects are risks that have a threshold value below which no adverse effects occur (Bazarzhapov et al. 2023). The non-carcinogenic health risk of the water sources was estimated using the reference (safe) dose (RfD) of exposure. RfD is the maximum tolerable dose of chemicals for humans through daily exposure that does not cause adverse effects in a human population, including sensitive subgroups (Bazarzhapov et al. 2023). The HQ was used to characterise the non-carcinogenic adverse health effects caused by exposure to heavy metals and CN using Eq. (7).

7 $$\mathrm{HQ}=\mathrm{CDI}/\mathrm{RfD}$$

where CDI (chronic daily intake) is the oral dose of heavy metals and CN present in the drinking water (mg/kg-day). The RfD (oral) values in mg/kg-day of the chemical substances analysed in this study are Fe (0.7), Mn (1.4 × 10^–1^) (Javed and Usmani 2016), Cd (0.5) (Mohammadi et al. 2019), Hg (3.0 × 10^–4^) (Chang et al. 2014), and CN (3.0 × 10^–3^) (USEPA 2010a, b). CDI was calculated as follows:

8 $$\mathrm{CDI}={C}_{m}\times W/{B}_{W}$$

where *C*_*m*_ is the concentration of the heavy metal or CN in the water sources; *W* is the average amount of water consumed per day by an adult (2.0 L), and *B*_*W*_ is the average weight of an adult (70.0 kg). When HQ < 1.0, adverse health effects are unlikely to be experienced, but when HQ ≥ 1.0, there is a potential for non-carcinogenic health effects (Al-Saleh et al. 1999).

The hazard index (HI) was used to assess the potential risk of the combined adverse health effects of individual components of a mixture of heavy metals and CN in the water sources. The HI was calculated as the sum of HQ (EPA 1986):

9 $$\mathrm{HI}=\sum \mathrm{HQ}$$

Classification of HI is as follows (Wu et al. 2010): HI < 0.1 is low risk; 0.1 ≤ HI < 1.0 is low to moderate risk; 1.0 ≤ HI < 5.0 is moderate risk; 5.0 ≤ HI < 10.0 is high risk; and HI > 10.0 is very high risk. Overall, if HI < 1.0, it is assumed that chronic risk is unlikely to happen, while HI ≥ 1.0 means there is a possibility for non-cancer risks to occur (Cao et al. 2015).

#### Carcinogenic risk analysis

The carcinogenic risk (CR) is assessed as the incremental probability of a person developing cancer over a lifetime of 70 years due to a 24-h exposure to a potential carcinogen (Salihu et al. 2019). The CR was calculated following Eq. (10):

10 $$\mathrm{CR}=\mathrm{CDI}\times {\mathrm{CSF}}_{\mathrm{o}}$$

where CSF_o_ is the oral cancer slope factor (mg/kg-day). CSF_o_ of Cd is 6.1 mg/kg-day (Mohammadi et al. 2019). Hg, Fe, Mg, and CN do not have CSF_o_ because they are not considered to cause cancer. The permissible risk limits are 1.0 × 10^−6^ < CR < 1.0 × 10^−4^ (Cao et al. 2015; USEPA 2005).

### Statistical analysis

#### Test for normality of water quality data

Before applying multivariate statistical techniques to the water quality dataset, a normality assessment was performed for all parameters using the Shapiro–Wilk test (see Table S5 in the Supplementary Material). The Shapiro–Wilk test was selected because it is one of the most robust and reliable tests for detecting deviations from normality, particularly in small sample sizes of up to 50 (Khatun 2021; Korkmaz and Demir 2023). The outcome of the test informed the choice of multivariate methods used in subsequent analysis. Elements with *p* ≤ 0.05 are considered to deviate significantly from normal distribution.

#### Correlation analysis

Correlation analysis was used to understand the relationship between the water quality parameters. Based on the results from the normality test, the Spearman correlation analysis was applied as it is suitable for data which does not assume normality (Schober et al. 2018). Cu and Pb were excluded from statistical analysis because all measurements were below detection limits. Mn exhibited 80.0% censored values and was therefore analysed using non-parametric tests only.

#### Principal component analysis and hierarchical cluster analysis

Principal component analysis (PCA) was applied using IBM-SPSS version 23 software to examine the compositional patterns of the water sources and identify the elements that influence each of them. The PCA with varimax rotation was used to gather extensive information about the sort of natural and anthropogenic sources responsible for enriching pollutants and their mobility in the water. The variance contribution rate was used to calculate the principal component scores. The weight and the composite score were obtained afterwards.

Hierarchical cluster analysis (HCA) was used to determine the groupings by determining the sampling locations, which contained similar amounts of the parameter analysed. It allows for a more accurate interpretation and comprehension of water quality (Selle et al. 2013). The HCA analysis generated a dendrogram or cluster tree as inter-sample similarities are ranked or linked in a dataset to form a cluster of samples.

## Results and discussion

### Physical characteristics of water quality

Turbidity, TDS, and EC were the three physical parameters analysed in this category. Table 1 presents the average values of the physical water quality characteristics of 20 water sources used for consumption in the Asankrangwa community in the Western Region of Ghana. A total of 5 SW or streams and 15 groundwater sources, representing 9 BH and 6 HDW, were analysed within 30.11 km of ASGM operation sites . The results revealed that the turbidity values of approximately 83.0% HDW sources, 80.0% SW sources, and 44.4% BG sources were higher than the WHO standard limit of 5 nephelometric turbidity units (NTU) for drinking water (WHO 2011). Extremely high values of 94.3 and 100.0 NTU were found in one HDW (HDW3) (about 2.83 km away from the ASGM sites) and the BH at the mining site (BHG), respectively. Additionally, 40.0% of the streams had exceptionally high turbidity values (41.1–52.4 NTU) as compared with WHO norms. Similar results have been found in streams within gold mining areas in Ogun State, Nigeria, ranging from 35.5–222.9 NTU, with higher levels in the raining season due to continuous contamination by organic and inorganic materials from mining-related activities (Olalekan et al. 2023). Turbidity leads to cloudiness of the water, which is generated by various particles, plankton, silt, and clay (APHA 2012; Apau et al. 2022; Rachmawati et al. 2022). Higher turbidity influences biological processes in streams, such as algae development, which can lead to a reduction in dissolved oxygen in the water (WHO 2011). This, in turn, makes the water foul and unhealthy, negatively affecting drinking water quality. It was observed during sampling that ASGM operations create a muddy environment at their sites, and the (contaminated) wastewater is discharged directly to nearby water bodies. Hence, the high turbidity in the water sources within the ASGM area is due to the transfer of dust particles and colloidal substances from the mining sites to the water bodies via leaching to groundwater, erosion, and dissolution of dust particles to the streams.

**Table 1:** Physical properties of the surface and underground water sources

Samples	Turbidity (NTU)	TDS (mg/L)	Conductivity (µS/cm)
BHG	100.0	157.7	351.7
BH1	1.5	250.2	413.8
BH2	2.0	88.7	151.0
BH3	14.5	211.7	358.6
BH4	2.1	71.3	198.0
BH5	5.8	87.5	147.8
BH6	2.1	85.6	145.9
BH7	1.7	243.6	428.8
BH8	10.1	87.9	150.9
HDW1	5.9	257.9	341.9
HDW2	5.2	97.1	169.2
HDW3	94.3	242.6	493.8
HDW4	3.4	73.9	125.8
HDW5	15.5	86.9	151.8
HDW6	3.0	220.4	157.4
SW1	41.1	77.5	131.3
SW2	52.4	113.1	192.9
SW3	11.1	138.4	158.8
SW4	2.9	187.8	387.4
SW5	7.1	145.8	221.5

*BH*, Borehole; *BHG*, borehole at mining site; *HDW*, hand-dug well; *SW*, surface water. The numbers 1 to 8 represent the different water sources

TDS should be below 1000.0 mg/L in drinking water (WHO 2011). Hence, the TDS of water sources in the ASGM study area meets the acceptable limit. Comparable results have been reported by a different study conducted for rivers (Pra, Offin, and Birim) within ASGM areas in Ghana, with TDS values ranging from 31.0 to 684.0 mg/L, which varied based on the locations of the water sources (Bessah et al. 2021). Studies within large-scale gold mining areas in Bibiani and Newmont Ghana concessions in the Western and Eastern Regions of Ghana also found similar results (Asamoah-Boateng 2009; Gyawu-Asante et al. 2017). Dukiya et al. (2023) found TDS in the range 42.9–119.0 mg/L in River Kpapi within gold mining areas in Minna, Nigeria. TDS values between 89.0 and 158.0 mg/L have been found in surface waters within gold mining areas in Ogun State, Nigeria (Olalekan et al. 2023). In their study, TDS was highest in the dry season, which was associated with concentration of dissolved organic matter and dissociated electrolytes entering the surface water. Hence, it can be inferred that gold mining activities do not generally increase the TDS levels in streams and groundwater sources above the acceptable level for drinking water. Rather, the TDS values are associated with the anthropogenic activities and geology of the catchment area (Adesakin et al. 2020).

The EC of the water samples meets the WHO requirements (< 1000.0 µS/cm). This means the waters contain low amounts of dissolved ionic substances or inorganic salts (Adesakin et al. 2020; Apau et al. 2022). EC values ranging from 49.3 to 1104.0 µS/cm have been found in rivers (Pra, Offin, and Birim) within ASGM areas in Ghana, with some values exceeding the WHO limit (Bessah et al. 2021). However, the proximity of the rivers to the ASGM sites is not known; hence, the high EC levels could be caused by other anthropogenic activities. Reported EC of water sources within large-scale gold mining areas, Asante Gold (Bibiani, Western region) and Newmont Ghana Gold Mining (Eastern Region), were within the range of the WHO standard limit (Asamoah-Boateng 2009; Gyawu-Asante et al. 2017). Sani et al. (2023) reported EC between 342.0 and 610.0 mg/L in groundwater in Meshegu, Niger State, Nigeria, while 67.0–186.0 mg/L were found in River Kpapi in Minna, Nigeria (Dukiya et al. 2023). EC values ranging from 150.0 to 225.0 mg/L have been found in surface waters in Ogun State, Nigeria, with EC levels higher until the end of the dry season and the beginning of the raining season, indicating the effect of anthropogenic activities (Olalekan et al. 2023). The EC values in some of the water samples in this work showed a significant rise because most samples were collected near the ASGM mining sites. However, this study has revealed that ASGM operations do not lead to high values of EC in drinking water sources.

### Chemical characteristics of water quality

Table 2 presents the chemical characteristics of the water sources analysed. The pH of 75.0% of the water sources is slightly acidic (< 6.5) and was below the WHO acceptable standard of pH 6.5–8.5 (WHO 2011). The results are comparable to the pH values reported by Gyawu-Asante et al. (2017) for water sources within Ghana’s Bibiani (Asante Gold) commercial gold mining area. Their results range from 5.8 to 6.6 pH units for groundwater compared with SW with pH from 6.6 to 7.1. The natural chemistry of the groundwater is controlled by the dissolution of the geological materials through which the water flows. Hence, the acidity in these water sources may be caused by the following factors: (i) Leachate draining through acid drainage (Naicker et al. 2003) from the ASGM sites, which seeps into the water body. (ii) The chemicals used in processing the mineral ores may produce acid effluence that seeps into groundwater or flows into nearby SW bodies. (iii) The reactions between the metal-rich rock minerals, water, and atmospheric oxygen may also cause acid drainage. The solubility of heavy metals at lower pH is high; hence, they are more toxic at lower pH. As a result, these water sources are dangerous for consumption.

**Table 2:** Chemical characteristics of the surface and ground water sources

Samples	pH	Fe^2+^ (mg/L)^a^	Mg^2+^ (mg/L)	Ca^2+^ (mg/L)	TH (mg/L)	Alkalinity (mg/L)	CN (mg/L)
BHG	5.8	0.24	40.0	694.0	734.0	105.0	3.0 × 10^–1^
BH1	6.1	0.16	120.0	2241.3	2361.3	15.0	1.0 × 10^–3^
BH2	5.9	0.31	60.0	732.0	792.0	18.0	4.0 × 10^–3^
BH3	7.3	0.43	100.0	1063.3	1163.3	90.0	1.0 × 10^–3^
BH4	5.5	0.15	40.0	1676.0	1716.0	14.5	3.0 × 10^–3^
BH5	6.3	0.10	80.0	2804.0	2884.0	20.0	4.0 × 10^–3^
BH6	6.0	0.08	40.0	2204.0	2244.0	21.4	0.0
BH7	6.0	0.10	80.0	2218.7	2298.7	18.0	0.0
BH8	6.5	0.60	40.0	700.0	740.0	65.0	0.0
HDW1	7.2	0.08	160.0	2160.0	2320.0	70.0	0.0
HDW2	6.5	0.15	60.0	1272.0	1332.0	35.0	7.0 × 10^–3^
HDW3	6.4	0.18	220.0	4728.0	4948.0	72.0	5.0 × 10^–3^
HDW4	6.1	0.20	40.0	445.0	485.0	25.0	3.0 × 10^–3^
HDW5	5.9	0.24	60.0	1060.0	1120.0	15.0	8.1 × 10^–2^
HDW6	5.4	0.15	100.0	950.0	1050.0	100.0	4.0 × 10^–3^
SW1	7.2	0.16	60.0	1540.0	1600.0	75.0	1.2 × 10^–2^
SW2	7.1	0.24	60.0	2436.7	2496.7	64.0	1.0 × 10^–3^
SW3	6.3	0.20	60.0	445.0	505.0	95.0	4.0 × 10^–3^
SW4	5.9	0.20	60.0	2744.0	2804.0	175.0	0.0
SW5	5.8	0.10	80.0	1030.0	1110.0	70.0	0.0

*BH*, borehole; *BHG*, borehole at mining site; *HDW*, hand-dug well; *SW*, surface water. The numbers 1 to 8 represent the different water sources. ^a^Used 2 decimal places, respectively, due to the low detection range

‘Total hardness (TH) measurements in the water sources ranged from 485.0 to 4948.0 mg/L. Based on TH categorisation, all the water sources fall within the very-hard group (TH > 180.0 mg/L). The highest permissible limit (HPL) of TH for drinking is 500.0 mg/L, and the most desired limit is 100.0 mg/L (WHO 2011). The TH of all the BH and streams was above the HPL, while 83.0% of the hand-dug wells had values higher than the HPL. TH is not harmful, but concentrations above 200.0 mg/L cause the formation of scum, while TH > 200.0 mg/L leads to the formation of calcium carbonate scales when the water is heated (WHO 2011). The results indicate that Ca^2+^ contributed more to the higher TH levels in the water sources than Mg^2+^. This may be due to the weathering of limestone, sedimentary rock, and calcium-bearing minerals, caused by the ASGM activities’.

The borehole at the mining site (BHG) contains a CN level that is 1.5 times higher than the Maximum Contaminant Level Goal (MCLG) of 0.2 mg/L (USEPA 2020). Consuming this water source, even for relatively short periods, would expose miners to serious health risks. These include rapid breathing, neurological effects like tremors, and chronic complications such as thyroid problems and other neurological issues (USEPA 2022). Except at BHG, CN concentrations in the SW and groundwater sources were below the MCLG or the detection limit. The lower cyanide (CN) levels are comparable to the results reported by Gyawu-Asante et al. (2017) from the Bibiani (Asante Gold) commercial gold mining areas in Ghana. They found total CN levels between about 2.0 × 10^–3^ and 4.0 × 10^–2^ mg/L in groundwater sources, while surface water (SW) ranged from 8.0 × 10^–3^ to 2.0 × 10^–2^ mg/L. A study by Asamoah-Boateng (2009) also reported CN levels between 1.0 × 10^–2^ and 5.0 × 10^–2^ mg/L from the Newmont Ghana gold mining commercial mining area. The findings suggest that ASGM operations do not result in significant CN contamination of water bodies in mining areas. This may be attributed to the following reasons: first, the leaching process is more controlled, thereby minimising spillage from the tailings into the environment; second, CN spills in surface water (SW) are known to break down rapidly (Earthworks 2023); additionally, the CN formed metal–cyanide complexes, which readily sorb to soils with high anion exchange capacities such as clays (Dzombak et al. 2006). However, groundwater contaminated with CN can persist for a long time, polluting drinking water aquifers (Earthworks 2023). This accounted for the higher level of CN in the BHG water source.

The alkalinity levels of the groundwater and SW sources were below the threshold limit of < 500.0 mg/L for drinking water. However, an average of 350.0 mg CaCO_3_/L has been recommended (Akter et al. 2016). High alkalinity of 175.0 mg/L was found in SW4, and the lowest value of 15.0 mg/L was detected in a few groundwater sources (BH1, BH4, and HDW5). Similarly, alkalinity values less than 350.0 mg CaCO_3_/L were found in water sources within the Bibiani large-scale gold mining areas (Gyawu-Asante et al. 2017). High alkalinity in water results from the precipitation and leaching of organic matter (Rakotondrabe et al. 2017; Apau et al. 2022). This means ASGM and mining operations do not lead to high levels of alkalinity in water in mining communities.

### Piper trilinear diagram

**Fig. 2:**
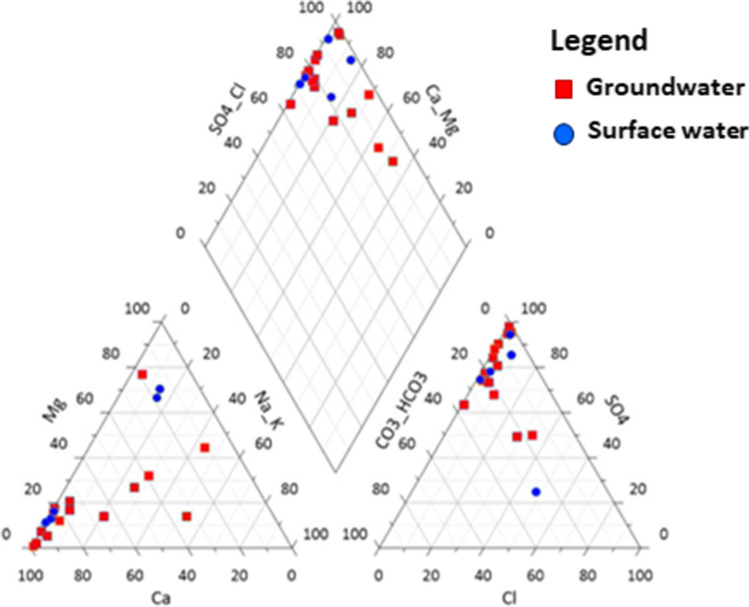
Hydrochemical facies shown on Piper’s trilinear diagram, including dominant anions, cations, and water samples classification

Figure 2 presents a Piper trilinear diagram, which was useful for analysing the chemical relationships among the water sources in communities within the ASGM sites. The concentration of anions and cations was normalised to 100. Figure 2 indicates that the water sources in the communities within the ASGM areas are dominated by Ca^2+^–Mg^2+^–Na^+^–HCO_3_^−^ and Ca^2+^–Mg^2+^–Na^+^–HCO_3_^−^–SO_4_^2−^. The preponderance of Na, Ca, and Mg ions varies, likely resulting from the dissolution of silicates. The reduction of sulphate (through the oxidation of sulphides) and root zone material by microbial activities may have produced the HCO_3_ ions. The complete chemical properties of the water sources used in Fig. 2 are presented in Table 3.

**Table 3:** Cations and anions characteristics of the surface and underground water sources

Samples	Distance from site (Km)	Cl^−^ (mg/L)	HCO_3_ (mg/L)	SO_4_ (mg/L)	Na^+^ (mg/L)	K^+^ (mg/L)
BHG	0.00	25.0	2.1	202.8	43.0	14.0
BH1	2.97	43.7	6.0	18.4	120.0	37.0
BH2	30.11^a^	26.2	6.8	80.4	27.0	12.3
BH3	2.40	38.7	1.0	238.0	437.0	78.4
BH4	2.46	7.5	2.3	36.0	240.0	64.0
BH5	1.56	6.3	5.2	9.3	237.0	40.0
BH6	2.19	5.0	11.9	7.5	700.0	71.0
BH7	1.30	8.8	7.4	1.9	520.0	68.2
BH8	2.68	6.3	6.3	8.4	227.0	7.9
HDW1	3.01	11.3	2.1	9.0	105.0	34.4
HDW2	2.78	13.8	1.0	65.0	54.0	60.0
HDW3	2.83	8.8	4.6	293.0	512.0	40.0
HDW4	6.52	18.7	1.5	124.0	124.0	70.0
HDW5	3.05	179.9	1.0	157.0	320.0	93.5
HDW6	4.18	20.0	7.9	204.0	402.0	6.0
SW1	4.65	23.7	11.0	187.0	714.0	11.3
SW2	3.11	11.3	2.7	3.0	500.0	14.7
SW3	2.81	6.3	3.1	3.9	227.0	54.0
SW4	6.44	12.5	1.0	114.0	100.0	32.2
SW5	1.32	117.5	1.5	301.0	37.0	11.8

From the ternary plot of cations (left triangle), most of the ions are clustered around the Ca^2+^ pole, with a few ions towards the Mg^2+^ pole. This is observed for both SW and groundwater; however, the concentrations in the groundwater are greater than those in the SW. The high concentration of calcium and the differences in the observed concentrations in the water sources could be due to the degree of weathering of primary mineral sources and the rock mineralogy at different sites. The cations’ dominance in the water sources is in the order Ca^2+^ > Mg^2+^ > Na^+^ > K^+^.

The ternary plot of anions (right triangle) suggested a high level of sulphate ions present in both groundwater and SW samples and very low concentrations of bicarbonate ions, based on the position of the clusters. The high sulphate concentration could be caused by the influence of acid mine drainage from the ASGM areas.

As shown in the diamond plot (rectangular diagram), the cluster of ions evolved towards the Ca-Mg and the SO_4_-Cl poles and is concentrated in the Ca-SO_4_ region. The low potassium levels in the water sources may be because of their low geochemical mobility. This observation can be made for both the streams and the groundwater. Figure 2 revealed that the area contains considerable sulphides, particularly arsenopyrite and pyrite. Their oxidation could lead to SO_4_^2−^ ions in the water sources. The Cl^−^ in the water sources may be due to anthropogenic pollution and intrusive rocks characteristic of granitoids in the area. From the position of the ions in the diagram, it could be concluded that the water types are of permanent hardness.

### Heavy metal concentrations

Table 4 presents the concentration of heavy metals in the water sources within the ASGM communities. The primary natural sources of heavy metal transport to water bodies are soil erosion, weathering of mineral-bearing rocks, and dissolution of water-soluble salts (Varol et al. 2012). Cd levels in the water sources were far above the GSA (2013) and WHO (2011) standard limit of < 3.0 × 10^–3^ mg/L, and the MCLG of < 5.0 × 10^–3^ mg/L (USEPA 2022). That is about 4.7–23.0 times higher than the GSA (2013) and WHO (2011) limit or 2.8–13.8 times above the MCLG. The high levels of Cd may be attributed to its persistence and accumulation in sediments and organisms, a general trend reported for heavy metals in water (Cui et al. 2021). Cd is carcinogenic and causes kidney damage in humans, with a biological half-life of 10.0–35.0 years (WHO 2011). The distance from the ASGM sites did not impact the concentration of Cd in the water sources. The highest concentration of Cd (6.9 × 10^–2^ mg/L) was found in SW5, which is closer (i.e. 1.32 km) to the mining site and may be caused by erosion. The results revealed that the consumption of water sources within the ASGM communities would be detrimental to human health due to the very high levels of Cd. On the contrary, Bessah et al. (2021) found low levels of Cd (1.0 × 10^–3^–1.4 µg/L) in rivers (Pra, Offin, and Birim) within ASGM areas in Ghana, which were within the acceptable limit for drinking water quality. These reported concentrations are lower than those found in this study for the streams (1.4 × 10^–2^–6.9 × 10^–2^ mg/L). Cobbina et al. (2015) found 5.3 × 10^–1^ and 2.3 × 10^–2^ mg/L of Cd in two rivers, Nangodi and Tinga, respectively, in ASGM areas in Northern Ghana, which were above the MCLG (USEPA 2022) and GSA (2013) or WHO (2011) acceptable limits. However, the distance from the rivers to the ASGM sites was not reported for comparison to this current work. Very high concentrations of Cd between 0.2 and 0.8 mg/L have been found in stream waters within gold mining areas in the Ecuadorian Amazon (Galarza et al. 2023). High levels of Cd have also been reported in water sources within gold mining areas in Nigeria, such as: 0.0–5.0 × 10^–3^ in groundwater and 1.0 × 10^–3^–1.2 × 10^–2^ in Meshegu (Sani et al. (2023), and 2.0 × 10^–2^–6.0 × 10^–2^ in River Kpapi in Minna (Dukiya et al. 2023).

**Table 4:** Concentration of heavy metals identified in the surface and underground water sources

Samples	Distance from site (Km)	Fe (mg/L)	Cd (mg/L)	Hg (mg/L)	Cu (mg/L)	Mn (mg/L)	Pb (mg/L)
BHG	0.00	4.8 × 10^–2^	4.0 × 10^–2^	7.2 × 10^–1^	BDL	2.2 × 10^–1^	BDL
BH1	2.97	BDL	3.1 × 10^–2^	1.5 × 10^–2^	BDL	BDL	BDL
BH2	30.11^a^	2.5 × 10^–2^	3.8 × 10^–2^	2.7 × 10^–2^	BDL	BDL	BDL
BH3	2.40	1.3 × 10^–2^	4.5 × 10^–2^	1.0 × 10^–3^	BDL	BDL	BDL
BH4	2.46	4.9 × 10^–2^	5.3 × 10^–2^	3.0 × 10^–3^	BDL	BDL	BDL
BH5	1.56	8.9 × 10^–2^	5.9 × 10^–2^	9.0 × 10^–3^	BDL	BDL	BDL
BH6	2.19	7.9 × 10^–2^	4.2 × 10^–2^	1.9 × 10^–2^	BDL	BDL	BDL
BH7	1.30	4.1 × 10^–2^	3.1 × 10^–2^	4.0 × 10^–4^	BDL	BDL	BDL
BH8	2.68	7.1 × 10^–2^	4.3 × 10^–2^	2.1 × 10^–1^	BDL	BDL	BDL
HDW1	3.01	BDL	5.1 × 10^–2^	3.2 × 10^–2^	BDL	5.0 × 10^–3^	BDL
HDW2	2.78	1.1 × 10^–2^	3.0 × 10^–2^	4.0 × 10^–4^	BDL	BDL	BDL
HDW3	2.83	4.2 × 10^–2^	4.9 × 10^–2^	1.0 × 10^–3^	BDL	BDL	BDL
HDW4	6.52	BDL	4.1 × 10^–2^	5.5 × 10^–2^	BDL	BDL	BDL
HDW5	3.05	4.2 × 10^–2^	5.1 × 10^–2^	2.0 × 10^–3^	BDL	BDL	BDL
HDW6	4.18	5.2 × 10^–2^	4.2 × 10^–2^	4.0 × 10^–3^	BDL	BDL	BDL
SW1	4.65	9.2 × 10^–2^	5.2 × 10^–2^	1.3 × 10^–2^	BDL	2.4 × 10^–3^	BDL
SW2	3.11	8.7 × 10^–2^	3.5 × 10^–2^	7.2 × 10^–2^	BDL	9.0 × 10^–3^	BDL
SW3	2.81	7.8 × 10^–2^	1.4 × 10^–2^	5.3 × 10^–2^	BDL	BDL	BDL
SW4	6.44	6.0 × 10^–2^	5.3 × 10^–2^	3.1 × 10^–2^	BDL	BDL	BDL
SW5	1.32	4.3 × 10^–2^	6.9 × 10^–2^	1.0 × 10^–3^	BDL	BDL	BDL

^a^control point. *BDL*, below detection limit; *BH*, borehole; *BHG*, borehole at mining site; *HDW*, hand-dug well; *SW*, surface water. Detection limit (DL) of Mn = 0.02 mg/L; DL of Cu = 0.009; DL of Pb = 0.082 mg/L; DL of Fe = 0.03 mg/L

In terms of Hg concentrations (1.0 × 10^–3^–7.2 × 10^–1^ mg/L), the BH at the ASGM site (BHG) contained an extremely high level (7.2 × 10^–1^ mg/L); about 361 times greater than the MCL of 2.0 × 10^–3^ mg/L (USEPA 2022), or 722 times higher than the GSA recommended level of 1.0 × 10^–3^ mg/L (GSA 2013). Approximately 78.0% of the BH contained Hg levels above the MCL and GSA limits, while 50.0% and 67.0% of HDW were above the MCL and GSA recommended limits, respectively. Generally, besides BHG, the streams contained higher Hg concentrations than the groundwaters, with 80.0% of the streams containing Hg levels above the MCL and GSA limits.

According to the WHO, Hg concentration in drinking water sources is below 0.0005 mg/L (WHO 2011). This means that the high Hg levels in the water sources are caused by the discharge of contaminated tailings from ASGM activities. Besides BHG, which has a far higher Hg level, there is no indication of a direct relationship between Hg concentrations and the distance to the mining site. The reason is that Hg contamination of rivers is caused by erosion and sedimentation, as most of the ASGM operations are carried out along riverbanks. Pollution of groundwater sources, on the other hand, is caused by the infiltration or permeation of contaminants through the soil into the water (Bhalla et al. 2012; Lone et al. 2012), and in this case, the mining-contaminated leachate. Land steepness and soil features such as texture and permeability affect erosion and leaching (Amano et al. 2020; Nguyen et al. 2011). However, such factors were not investigated in the present work. Hence, further studies could be conducted in this area to understand how they influence and interact with pollutant distribution and distance from the ASGM sites.

High levels of Hg (1.0 × 10^–2^–2.0 × 10^–2^ mg/L) and Cd levels (2.0 × 10^–3^–1.1 × 10^–2^ mg/L have been found in River Samre in the Wassa Amenfi West District of the Western Region of Ghana. These were attributed to the activities of a timber and plywood company close to the river (Nkoom et al. 2013). This river is in the same district as the present study; hence, the ASGM activities may have contributed to these high contaminations. Hg concentrations in rivers (Pra, Offin, and Birim) within the ASGM catchment in Ghana reported by Bessah et al. (2021) ranged from 5.0 × 10^–3^–7.6 μg/L, with 3.0% of the locations exceeding the WHO standard for drinking water (WHO 2011). The Hg level in SW5 (1.0 × 10^–3^ mg/L) fell within this range. In the study by Cobbina et al. (2015), 3.8 × 10^–2^ and 6.4 × 10^–2^ mg/L of Hg were found in rivers Nangodi and Tinga, respectively, in northern Ghana (GSA 2013; USEPA 2022; WHO 2011). The Hg levels in both rivers were above the MCL and GSA/WHO acceptable standard for drinking water. Hg pollution of the rivers was attributed to the washing of gold-bearing ores in the ASGM area and the flow of Hg-laden wastewater from the washing bay to the rivers. Water sources near ASGM activities contained high Hg concentrations. Casso-Hartmann et al. (2022) reported Hg concentrations in drinking water sources within ASGM areas in Colombia ranging from 1.7 × 10^–1^ to 9.7 × 10^–1^ μg/L, with total Hg levels in 55.6% of the sites based on individual measures exceeding the maximum Hg threshold of 1.0 × 10^–3^ mg/L. Hg levels between about 1.0 × 10^–3^–3.0 × 10^–3^ mg/L were found in stream waters in the Ecuadorian Amazon, with 7.0% of the water sources exceeding the MCL for drinking water quality (Mestanza‑Ramón et al. 2023). It must be emphasised that these studies covered Hg pollution of only river streams (surface water). The present work included underground water Hg pollution and the distance to the water sources. Sani et al. (2023) found high Hg concentrations up to 0.1 and 1.1 mg/L in ground and surface waters, respectively, in Meshegu, Nigeria, and high values between 0.1 and 2.0 mg/L have been found in surface waters in gold mining catchments in Ogun State, Nigeria, which were attributed to long-term build-up in the sediment of the river and its tributaries (Olalekan et al. 2023). One important consideration is that, unlike groundwater, the concentration of heavy metals in streams is influenced by the time and magnitude of the flow. Drier months with slower flows are found to have higher levels of Hg (Loza del Carpio and Ccancapa Salcedo 2020).

The concentrations of Cu and Pb in all the water sources were below the method detection limits (MDL) of 9.0 × 10^–3^ and 8.2 × 10^–2^ mg/L, respectively. Mn concentration in most water sources was below the MDL of 2.0 × 10^–2^ mg/L, except BHG, HDW1, SW1, and SW2 (which contain values less than the WHO’s stated recommendation of 0.4 mg/L). Fe concentrations in the water sources were lower than the WHO’s 0.3 mg/L limit. Fe in three groundwater sources within 6.52 km of the ASGM sites was below the detection limit of 3.0 × 10^–2^ mg/L. High levels of Mn and Fe are toxic to human health and aquatic life, but in low amounts, they are essential for metabolic activities (Dey et al. 2021; Li et al. 2014).

Contrary to this study, reported values of Mn in waters within a commercial gold mining area ranged from 0.1 to 7.2 × 10^–1^ mg/L in surface water and groundwater sources, respectively (Gyawu-Asante et al. 2017). Concentrations of Cu and Pb found in rivers (Pra, Offin, and Birim) within ASGM areas in Ghana (Bessah et al. 2021) ranged from 4.0 × 10^–2^–403.8 μg/L to 2.0 × 10^–2^–48.5 μg/L, respectively. They found Cu levels at all locations within the acceptable limit for drinking water quality, while the level of Pb in 30.2% of the river locations was higher than the WHO’s 1.0 × 10^–2^ mg/L limit for drinking water (WHO 2011). Cobbina et al. (2015) reported 0.3 and 3.1 × 10^–2^ mg/L of Pb in rivers Nangodi and Tinga, respectively, in northern Ghana, which were above the WHO (2011) limit. Fe levels exceeding 0.3 mg/L will impair the flavour and appearance of drinking water (WHO 2011), although there is no recommended value. Reported values of Fe in surface water and groundwater sources within a large-scale gold mining area were between 1.0 × 10^–2^ and about 3.0 mg/L (Asamoah-Boateng 2009; Gyawu-Asante et al. 2017), and up to 4.3 × 10^–1^ mg/L have been found in rivers (Pra, Offin, and Birim) within ASGM areas in Ghana (Bessah et al. 2021), which are higher than values found in this study within ASGM areas. Iron in water comes from weathered iron-rich formations and the leaching of effluents from disposed waste materials into the aquifer system (Egbueri et al. 2023). Mn may be caused by anthropogenic activities (Agbasi et al. 2023). However, Mn and iron Fe present in water sources within these mining communities may stem from two factors: the processing of the mineral feedstock and the extraction process, with the former being the primary contributor.

### Correlation analysis

**Fig. 3:**
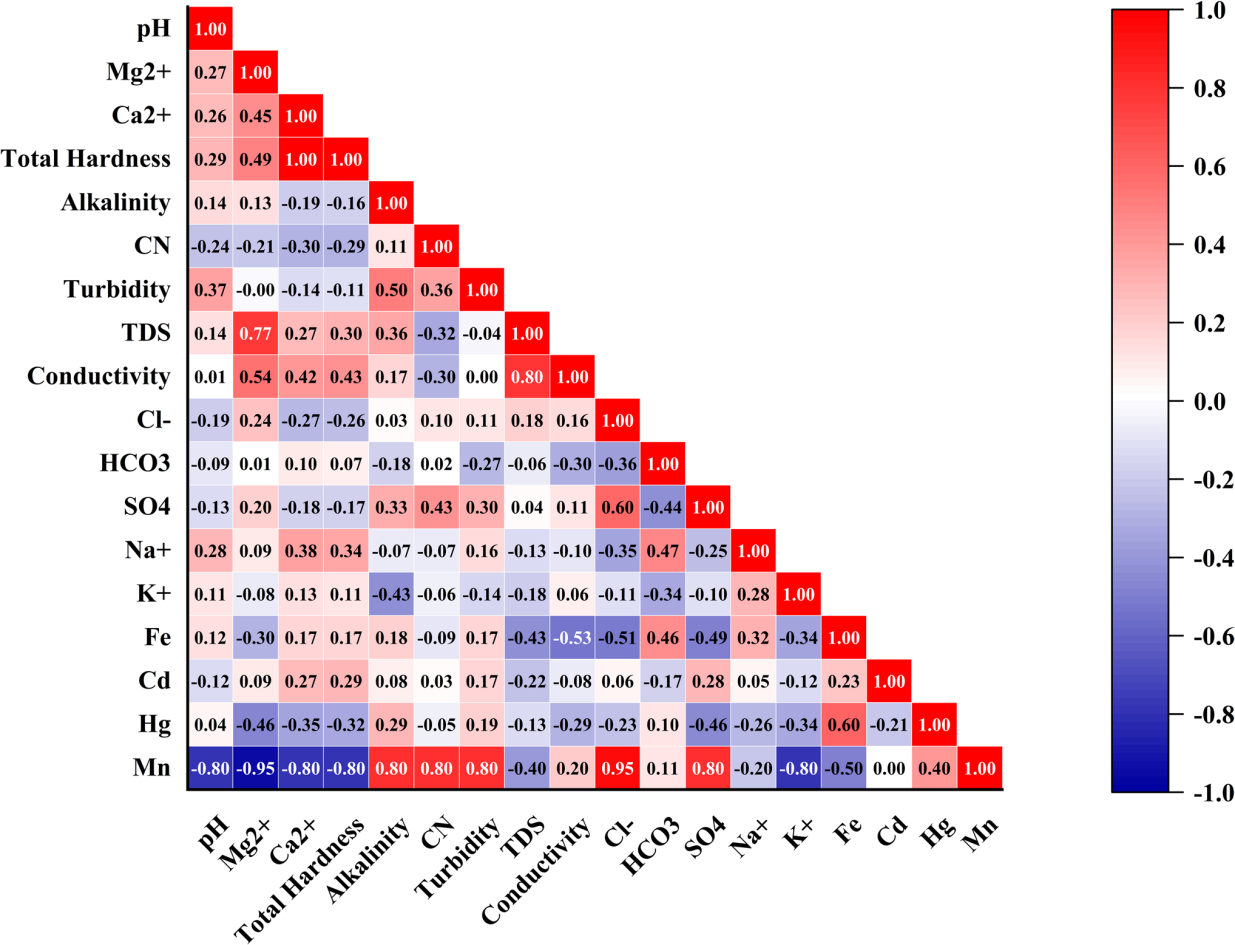
Spearman correlation matrices of water quality parameters

To show the relationship between the water quality parameters, Fig. 3 presents the Spearman correlation matrices. Each cell shows the Spearman correlation coefficient (*r*) between the two corresponding parameters, ranging from −1.0 (perfect negative correlation) to + 1.0 (perfect positive correlation). The colour bar on the right visually represents this scale, with bright red indicating a strong positive correlation and bright blue indicating a strong negative correlation. The correlation coefficients (*r*) were classified using commonly applied thresholds in environmental and water-quality studies (Mitryasova et al. 2023; Shakeri et al. 2025). Strong positive correlation: *r* ≥ 0.7, Moderate positive correlation: 0.4 ≤ *r* < 0.7, Weak positive correlation: 0.2 ≤ *r* < 0.4, Very weak or negligible correlation: − 0.2 < *r* < 0.2, Weak negative correlation: − 0.4 < *r* ≤  − 0.2, Moderate negative correlation: − 0.7 < *r* ≤  − 0.4, Strong negative correlation: *r* ≤  − 0.7.

The correlation matrix revealed several notable relationships among the measured parameters. Electrical conductivity (EC), total dissolved solids (TDS), Ca^2^⁺, Mg^2^⁺, and total hardness showed strong positive correlations. This indicates that increases in ions such as Ca^2^⁺, Mg^2^ increase the dissolved ionic content of water. These ions are responsible for the overall salinity and hardness of the water. The primary sources of these ions are geological (weathering of rocks and minerals), anthropogenic (agriculture, mining, urban runoff), and ion exchange processes (Gwira et al. 2024; Panghal et al. 2021).

Strong to moderate correlation was observed between total hardness, Ca^2^⁺, Mg^2^⁺, HCO₃⁻, Na⁺, SO₄^2^⁻. These correlations suggest that these ions frequently originate from the dissolution of similar minerals, primarily carbonates (calcite, dolomite), silicates, and evaporites (gypsum, halite), and are further influenced by ion exchange processes. For instance, the dissolution of calcite and dolomite releases Ca^2^⁺, Mg^2^⁺, and HCO₃⁻, while gypsum and halite contribute to SO₄^2^⁻ and Na⁺, respectively (Gugulothu et al. 2022; Sakram et al. 2018).

Moderate positive correlation was observed between SO₄^2^⁻ and Cl⁻. This suggests that these anions have a common source, which maybe because of anthropogenic inputs, the mining, or other sources such as irrigation return flow or fertiliser used in surrounding farms (Zhang et al. 2025). SO₄^2^⁻ is a common component of fertilisers such as ammonium sulphate and superphosphate (Spoelstra et al. 2021), and Cl⁻ may have been leached from common salt in soil and washed back into surface water (Ma et al. 2024).

Moderate positive correlation between Hg and Fe indicates that iron oxides, especially in mining-impacted areas, act as a sink for Hg, which controls its mobility. Hg may be remobilised under changing redox or pH conditions (de Souza et al. 2024; Manceau et al. 2018). pH exhibited weak to moderate negative correlations with SO₄^2^⁻, indicating that slightly more acidic conditions may be associated with the oxidation of sulphides, which are common in mining areas, releasing sulphate ions and releasing protons (H⁺), which acidify the water and lower pH (Lindsay et al. 2015; Whaley-Martin et al. 2023).

### Principal component analysis

**Fig. 4:**
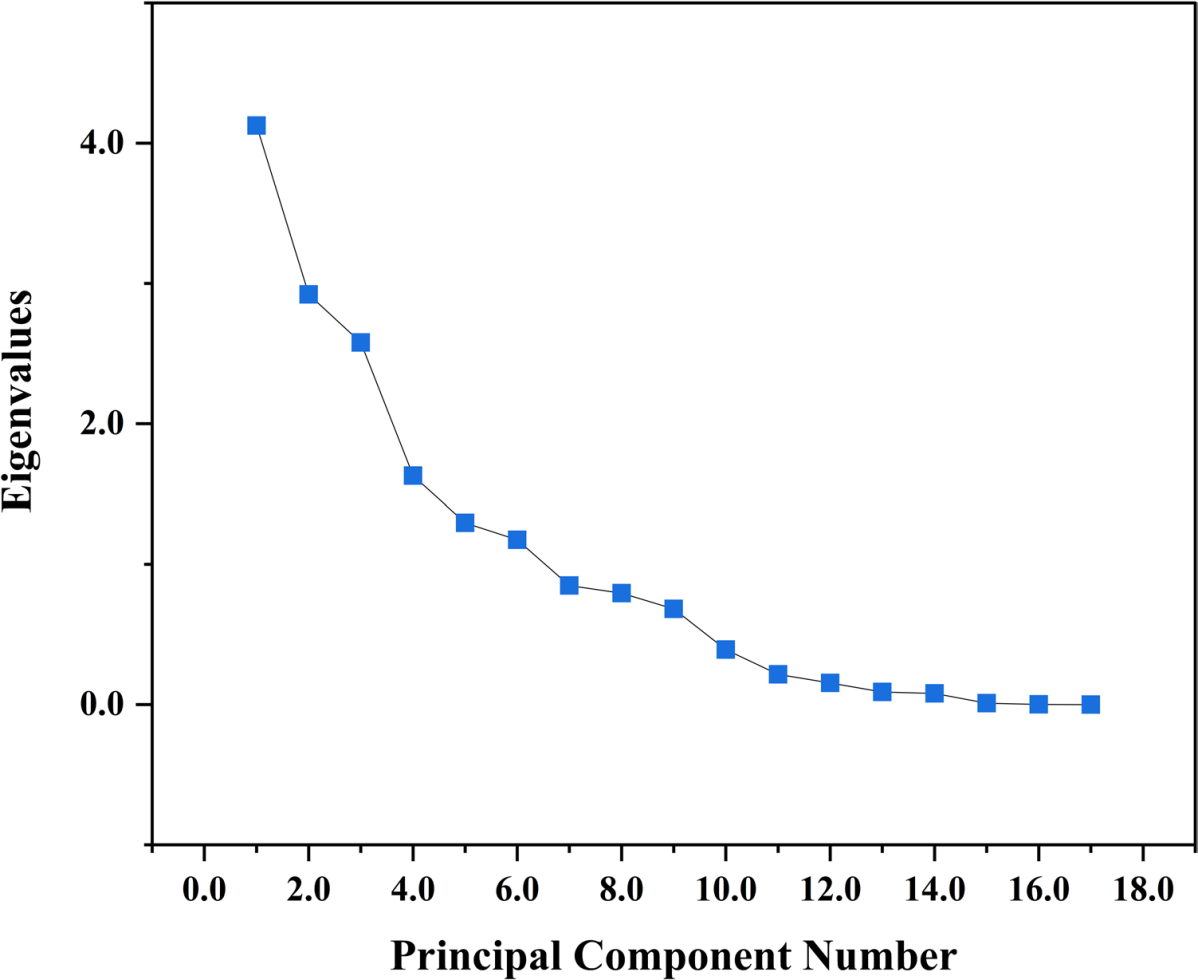
PCA scree plot

The analysis included all measured physico-chemical variables and detected heavy metals. Each variable was standardised using z-scores prior to PCA to remove the effects of differing measurement scales. The high multicollinearity observed in the correlation analysis makes the PCA an effective method for interpretation. The scree plot in Fig. 4 showed a distinct elbow after the third component, indicating that additional components contributed only marginal variance. In addition, only the first three components had eigenvalues greater than 2; therefore, three principal components (PC1, PC2, and PC3) were retained for interpretation. The principal component loadings, eigenvalues, percentage variance, cumulative eigenvalue, and cumulative percentages for the different water quality parameters are summarised in Table 5.

**Table 5:** Rotated component matrix of five-component model for physicochemical parameters

Parameter	Components		
	**PC1**	**PC2**	**PC3**
pH	0.2	−0.04	0.1
Mg^2+^	0.4	0.1	−0.1
Ca^2+^	0.4	−0.1	0.1
Total hardness	0.4	−0.1	0.1
Alkalinity	0.1	0.3	0.2
CN	−0.1	0.4	0.2
Turbidity	0.2	0.3	0.3
TDS	0.4	0.2	−0.1
Conductivity	0.4	0.3	−0.1
Cl^–^	−0.1	0.1	−0.3
HCO_3_	0.04	−0.4	0.3
SO_4_	0.01	0.2	−0.2
Na^+^	0.2	−0.3	0.3
K^+^	−0.02	−0.1	−0.3
Fe	−0.1	−0.2	0.5
Cd	0.1	−0.1	−0.1
Hg	−0.2	0.4	0.3
Eigenvalue	4.1	2.9	2.6
% of variance explained	24.3	17.2	15.2
Cumulative %	24.3	41.5	56.6

**Fig. 5:**
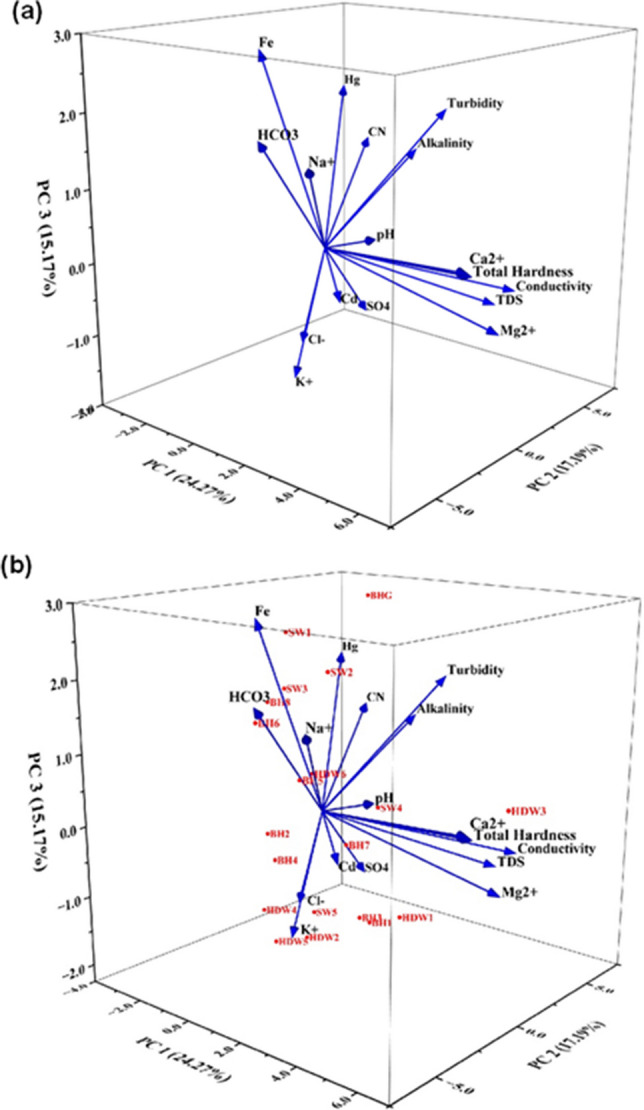
PCA plots: **a** loading plot and **b** biplot

The PCA revealed that the three principal components explained 56.6% of the total variance in the dataset (Fig. 5a). PC1 explained 24.3%, PC2 explained 17.2%, and PC3 explained 15.2% of the variability. PC1 showed high positive loadings for Mg^2^⁺ (0.4), Ca^2^⁺ (0.4), total hardness (0.4), TDS (0.4), and conductivity (0.4). PC2 had strong positive loadings from CN (0.4), Hg (0.4), turbidity (0.3), alkalinity (0.3), TDS (0.2), and conductivity (0.3). PC3 was characterised by a strong positive loading for Fe (0.5) and high contributions from HCO₃⁻ (0.3), Na⁺ (0.3), and turbidity (0.3).

The PCA biplots in Fig. 5b revealed spatial patterns among the three types of water sources in this study. Borehole (BH) samples clustered more around the central to negative PC2 region and near zero values on PC1. Hand-dug wells (HDW) were more dispersed but tended to fall on the negative side of PC1. Surface water (SW) samples mostly occupied higher values on PC2 and PC3. The biplot further revealed the relationship between sample positions with the corresponding variable vectors. Groundwater sources (BH and HDW) aligned more closely with Ca^2^⁺, Mg^2^⁺, TDS, conductivity, HCO₃⁻, K⁺, Cl⁻, while SW samples projected in the directions of turbidity, alkalinity, CN, Hg, and pH.

The PCA results show that three of the key components explain more than half of the total variability in the dataset. The distinct elbow in Fig. 4 confirms that using more than three components would contribute little additional insight, which may represent noise rather than meaningful structure (Ledesma et al. 2015; Reijer et al. 2024). PC1 contributed the largest share and primarily represents variations associated with major ions and mineralisation processes. The strong positive loadings for Ca^2^⁺, Mg^2^⁺, total hardness, TDS, and conductivity suggest that this component describes water–rock interaction processes affecting groundwater chemistry. This means this process greatly impacts water quality in the area. BH and HDW samples align with this axis, which shows that water samples from these sources are in contact with geological formations; therefore, their consistent ionic strength (Hailu and Haftu 2023; Wang et al. 2025).

PC2 contributes the second after PC1, and it reflects influence from surface contamination and anthropogenic input. The strong positive loading of CN, Hg, turbidity, alkalinity, TDS, and conductivity indicates water chemistry is influenced by surface runoff, land-use activities, and the input of suspended particulates. Particularly, Hg, CN, and turbidity point to the potential impacts of small-scale mining. The clustering of SW around these vectors indicates that these sources are highly impacted by surface activities as compared to groundwater. This shows that activities such as mining also influence the water chemistry (Gwira et al. 2024; Kumi et al. 2023).

PC3 contributes the least and captures redox and bicarbonate-driven processes, particularly in SW and a subset of BH samples. The strong loading of Fe on PC3 indicates the influence of redox-sensitive geochemical processes, where iron may be mobilised from subsurface materials under moderately reducing conditions. Its association with turbidity and bicarbonate suggests that this component represents particulate-associated metals and reactions that regulate iron solubility, including redox transitions and interactions between sediments and the water column (Kontny et al. 2021; Xia et al. 2022).

### Hierarchical cluster analysis

**Fig. 6:**
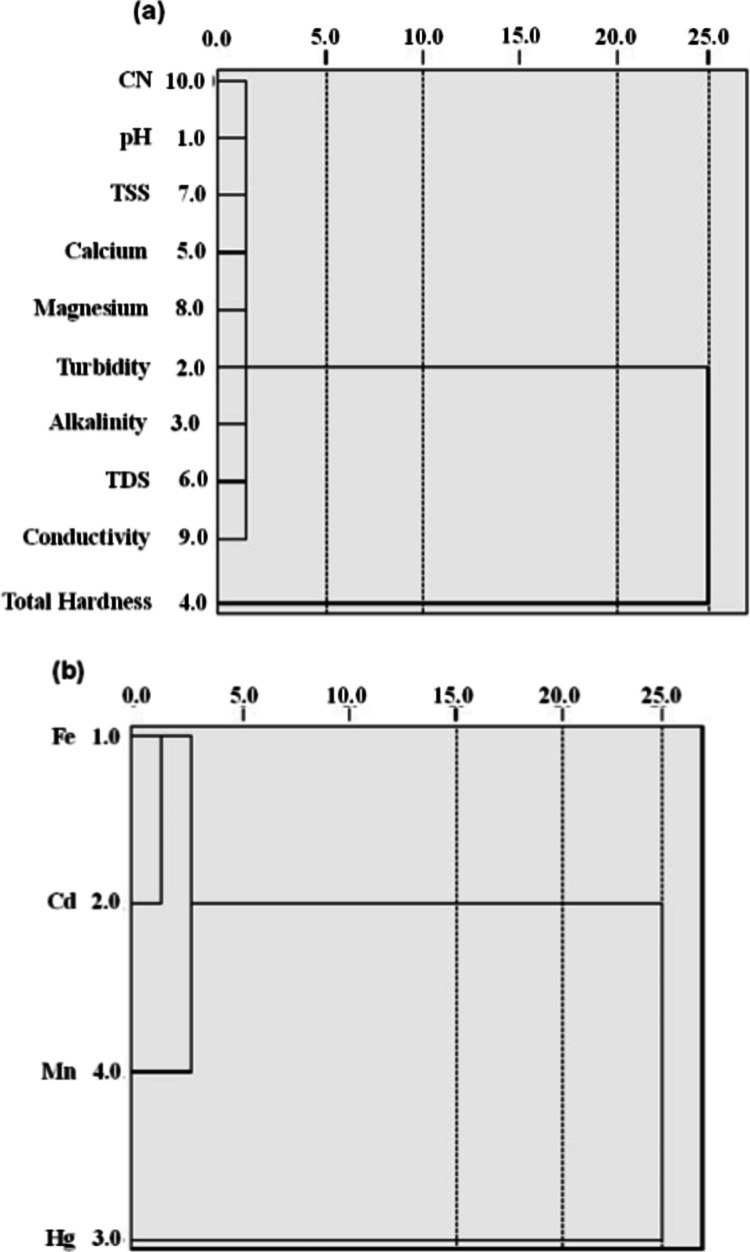
Hierarchical dendrogram of water sources: **a** physicochemical characteristics; **b** heavy metal concentrations. Average linkage (between groups) is used, and the rescaled distance cluster is combined

As shown in Fig. 6a, b, two clusters were created for physicochemical characteristics and heavy metal concentrations, which depend on their spatial similarities and variations. The first cluster of physicochemical parameters comprises turbidity and TH. The SWs in the study area were more polluted since particles in urban runoff and wastewater discharge from these miners can also contribute to increased turbidity of the SW. Turbidity in groundwater may also be attributed to inorganic particulate matter due to the weathering of rocks. A rise in the levels of cluster 1 members (Hg and Cd) in the heavy metal concentration negatively influences the level of cluster 2 members. This clarifies the wide separations of the two clusters. It indicates the influence of groundwater contamination via leaching and runoff from the ASGM sites.

### Water quality evaluation by water quality index

**Fig. 7:**
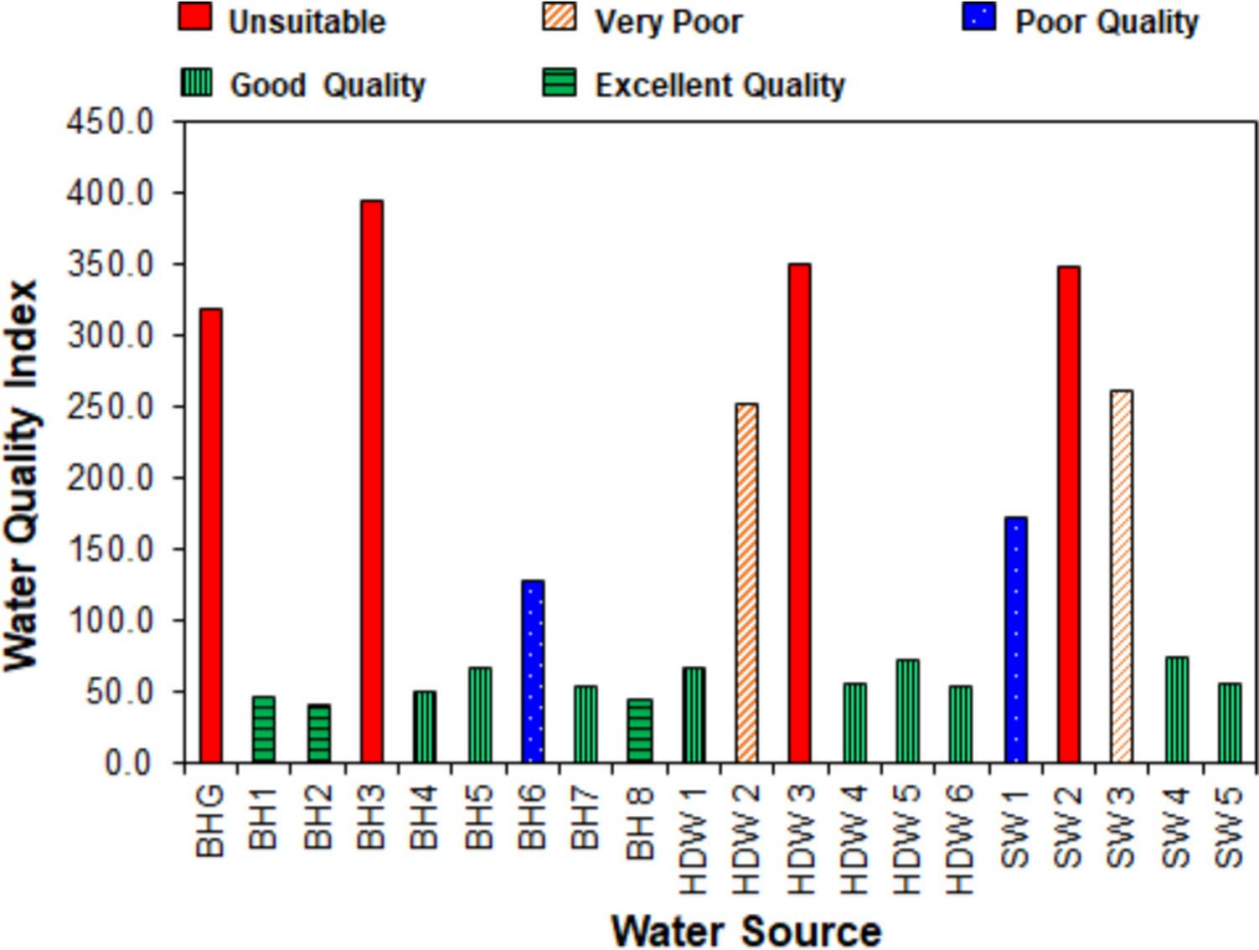
Water quality index of water sources in the ASGM areas

Figure 7 shows WQI values and the quality classifications of the water sources using the categorisations by Sahu and Sikdar (2008) based on physicochemical water quality parameters. The findings in Fig. 7 revealed that 20.0% of the water sources are unsuitable for consumption (WQI > 300.0), 10.0% are of very poor quality (WQI = 200.0–300.0), and poor quality (WQI = 100.0–200.0). Specifically, 20.0% of the streams are poor-quality water, 20.0% are very poor-quality water, and 20.0% are unfit for consumption. The high levels of turbidity and TH lead to the poor quality of the stream within 4.65 km of the mining site (SW1), the unsuitable quality of the water at 3.11 km (SW2), and the very poor quality of that within 2.81 km of the site (SW3) (see Table S6 in the Supplementary Materials).

For the groundwater sources, approximately 7.0% are of poor and very poor quality, and 20.0% are unfit for consumption. The BH at the mining site (BHG) is unsuitable for consumption due to the high levels of CN, turbidity, and TH. The bad quality of the other groundwater sources is also due to high concentrations of CN, turbidity, and TH (see Table S6). The WQI values demonstrate that the distance from ASGM sites to water sources impacts water quality. Besides BH2 and BH4, water sources within 3.11 km of the ASGM site are not of acceptable quality for consumption. Groundwater quality improves with increasing distance from contaminated sites (Amano et al. 2020; Rana et al. 2018), particularly beyond 2 km (Rana et al. 2018). The pollution of these waters originated from landfill sites, with leaching and infiltration being the primary mechanisms. Very high WQI values (2045.4–3087.5) have been reported by Dukiya et al. (2023) in River Kpapi in good mining catchments in Minna, Nigeria. WPI in the range 23.6–218.2 was found in groundwater in Alappad, a coastal area in Kerala, India, with only 7.0% belonging to ‘very poor’ quality. The poor quality was attributed to the concentrations of Cl^−^, Na^+^, TDS, and iron (Krishna and Achari 2024).

### Heavy metal pollution and evaluation indices of water

The values of the HPI rule were developed using Edet and Offiong’s (2002) three orders for drinking water. The average values were used to process the data, and a number of the average values were used to outline the various degrees of contamination. As a result, the HPI models proposed in this study are low (HPI < 400.0), medium (HPI = 400.0–800.0), and high (HPI > 800.0).

**Fig. 8:**
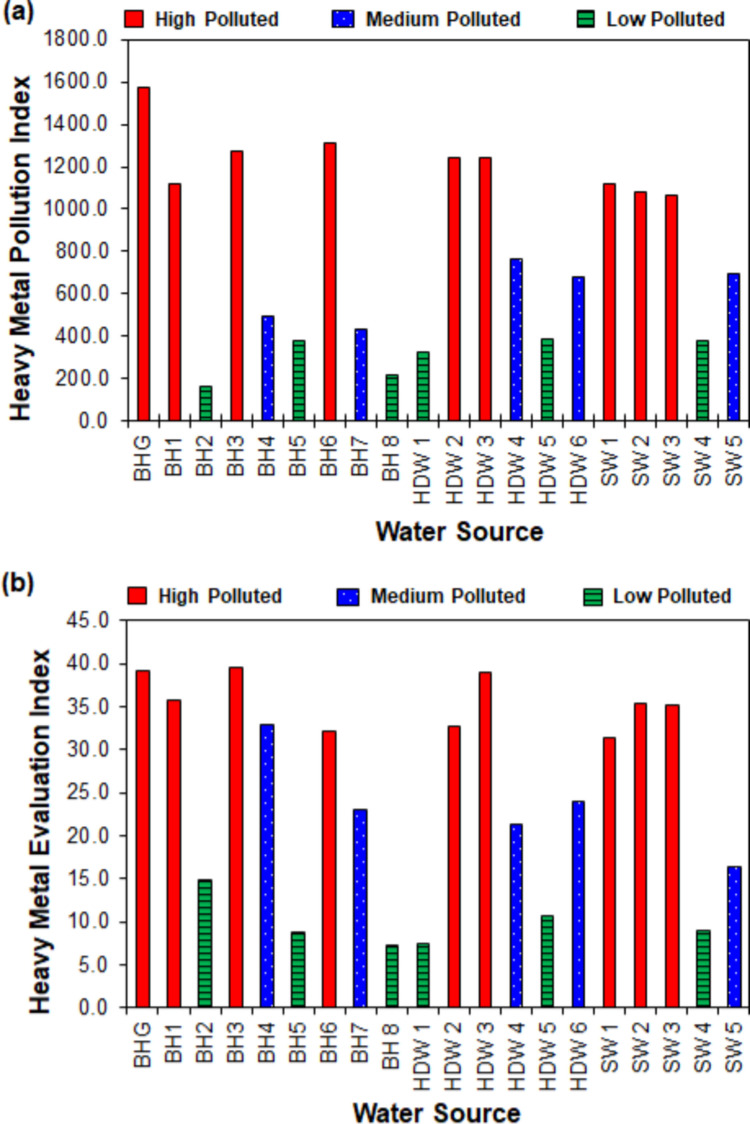
Heavy metal pollution indices of the water sources: **a** heavy metal pollution index; **b** heavy metal evaluation index HEI

From Fig. 8a, of all the water sources, 45.0% are within the highly polluted zone (1068.6–1571.9), 25.0% are medium polluted (432.1–767.2), and 30.0% are low contaminated (163.1–383.7). For the groundwater sources, 40.0% are in the highly contaminated category (1123.0–1571.9), about 27.0% are medium polluted (432.1–767.2), and nearly 33.0% are within the low polluted zone (163.1–383.7). It can be seen that 60.0% of all streams are within the highly contaminated zone (1068.6–1119.8), while 20.0% fall into the medium (692.7) and low (376.6) pollution zones. BHG at the ASGM mining site had the greatest HPI value of 1571.9, whereas the lowest HPI value (163.1) was found in BH2, about 30.11 km from the mining site. The high HPI values were a result of the high amounts of Hg and Cd in the waters near the mining sites, indicating that ASGM activities cause heavy metal pollution in water bodies. Amano et al. (2020) reported HPI values from 74.3 to 875.5 for water sources near a waste landfill site in the Kumasi Metropolis in Ghana, while Boateng et al. (2015) found HPI values in the range 319.2–688.1 for hand-dug wells within the Ejisu-Juaben Municipality, Ghana. A cumulative HPI score of 3570.0 has been reported for several rivers globally, including Armenia, Brazil, China, Germany, Greece, India, Iran, Japan, Latvia, Mexico, Nigeria, Pakistan, South Africa, Taiwan, and the USA (Kumar et al. 2019). HPI scores for river samples indicating low to critical pollution levels have been reported for different parts of the world, such as 16.0 and 142.0 in North Romania (Sur et al. 2022), 68.0 and 412.0 in West Romania (Moldovan et al. 2021), 10.0 and 40.0, 20.0 and 50.0, 86.0–915.8 in Turkey (Tokatli 2021; Tokatli et al. 2021; Varol and Tokatli 2023). The high HPI values (20 and 50) are attributed to agricultural activities (Tokatli 2021), while the 86.0–915.8 are due to farming practices and the abundance of heavy metals in the geological structure of the watershed (Tokatli et al. 2021).

The HEI was separated into three classes, like HPI, by multiplying the average value to categorise the pollution into (HEI < 16.0), medium (HEI = 16.0–30.0), and high (HEI > 30.0). The HEI results (Fig. 8b) displayed comparable trends with the HPI values throughout the numerous testing territories. The high-polluted class with HEI between 31.4 and 39.6 represented 45.0% of the water sources. The medium and low pollution categories with HEI scores of 16.4–24.0 and 7.3–14.8 represented 25.0% and 30.0% of the water sources, respectively. Although no direct link was established between the HPI and HEI values and the distances from ASGM sites to water sources, it was discovered that heavy metal contamination decreased beyond 12.68 km from the mining site, falling into the low-contaminated zone (see Table S7 in the Supplementary Materials). HEI scores have been reported for rivers in North Romania between 6.6 and 12.5 (Moldovan et al. 2021), 2.6 and 17.0 in West Romania (Sur et al. 2022), and HEI lower than 10.0, or ranging from 4.0 and 50.0 in rivers in Turkey in different studies (Tokatli 2021; Tokatli et al. 2021; Varol and Tokatli 2023).

HPI and HEI score categorisations of water resources within gold mining areas are presently limited in the literature. The differences between the reported HPI and HEI score categories of rivers in different countries indicate that heavy metal pollution is influenced by the type of activities and the geological composition of the study area.

### Human health risk evaluation

The degree of toxicity of poisonous substances such as heavy metals and cyanide to human health is linked to their daily intake. This study utilised ingestion through drinking water to analyse the non-carcinogenic and carcinogenic health risks of these substances.

#### Non-carcinogenic health risk

The non-carcinogenic analysis involved evaluating the CDI oral values (presented in Table S8), which gave average values of Cd as 1.2 × 10^−3^, 1.3 × 10^−3^, and 1.2 × 10^−3^ mg/kg-day for BH, HDW, and SW, respectively. The average values for Hg were 3.2 × 10^−3^, 4.5 × 10^−4^, and 2.2 × 10^−3^ mg/kg-day; and those for CN were 1.0 × 10^−3^, 4.8 × 10^−4^, and 9.7 × 10^−5^ for BH, HDW, and SW, respectively. The mean CDI values for Fe were 1.5 × 10^−3^, 1.1 × 10^−3^, and 2.1 × 10^−3^ mg/kg-day; and those of Mn were 6.1 × 10^−3^, 1.4 × 10^−4^, and 4.7 × 10^−4^ mg/kg-day for BH, HDW, and SW, respectively. Overall, the mean CDI oral values analysed for the water sources were found in the order of Hg > Mn > Fe > Cd > CN. Mean CDI oral or ingestion values of rivers in Romania were between 9.3 × 10^−4^ and 2.3 × 10^−1^ (Dippong et al. 2023). It has been found that water ingestion is one of the main entry routes of contaminants into the human body in contaminated areas (Emmanuel et al. 2022).

The HQ via oral/ingestion was evaluated (see Table S9 in the Supplementary Material). The influence of heavy metals and cyanide on non-carcinogenic harmful health risks was in the order of Hg > CN > Mn > Cd > Fe. The order of Hg contribution to the non-carcinogenic harmful health risk of the water sources was SW (12.3) > BH (10.7) > HDW (1.5), whereas that of CN was in the order of BH (1.7) > HDW (0.8) > SW (0.2). The contribution of Cd to non-carcinogenic harmful health risks of the water sources was in the order of HDW > SW > BH, while the order of contribution of Fe was SW > BH > HDW, and that of Mn was in the order of BH > SW > HDW. The HQs of Cd, Mn, and Fe for all the water sources were less than 1.0. This means that Cd, Mn, and Fe would be unlikely to have non-carcinogenic adverse health effects from consuming the water sources. Additionally, the HQs of Hg for BH3, BH4, BH5, BH7, HDW2, HDW3, HDW5, and SW5, as well as the HQs of CN for all water sources except BHG and HDW5, were less than 1.0. Hence, non-carcinogenic adverse health effects are unlikely to be experienced from Hg and CN in these water sources. However, HQ > 1.0 for CN in BHG and SW5, as well as Hg in BHG, BH1, BH2, BH6, BH8, HDW1, HDW4, and SW1 to SW4, suggests that there may be potential non-carcinogenic health effects associated with consumption. The results have shown that the type of water source impacts the non-carcinogenic harmful health risk effects of heavy metals and cyanide. Dippong et al. (2023) in their study of the risk of heavy metals in some rivers in Romania found mean HQ ingestion values in the range of 1.4 × 10^−2^ and 4.6 × 10^−1^, indicating that consumption of their studied rivers poses no harmful human health risk (HQ < 1.0). Similarly, HQ scores below 1.0 have been reported for trace-toxic metal accumulation rates in rivers in Turkey, presenting no harmful health risk through ingestion (Tokatli 2021).

**Fig. 9:**
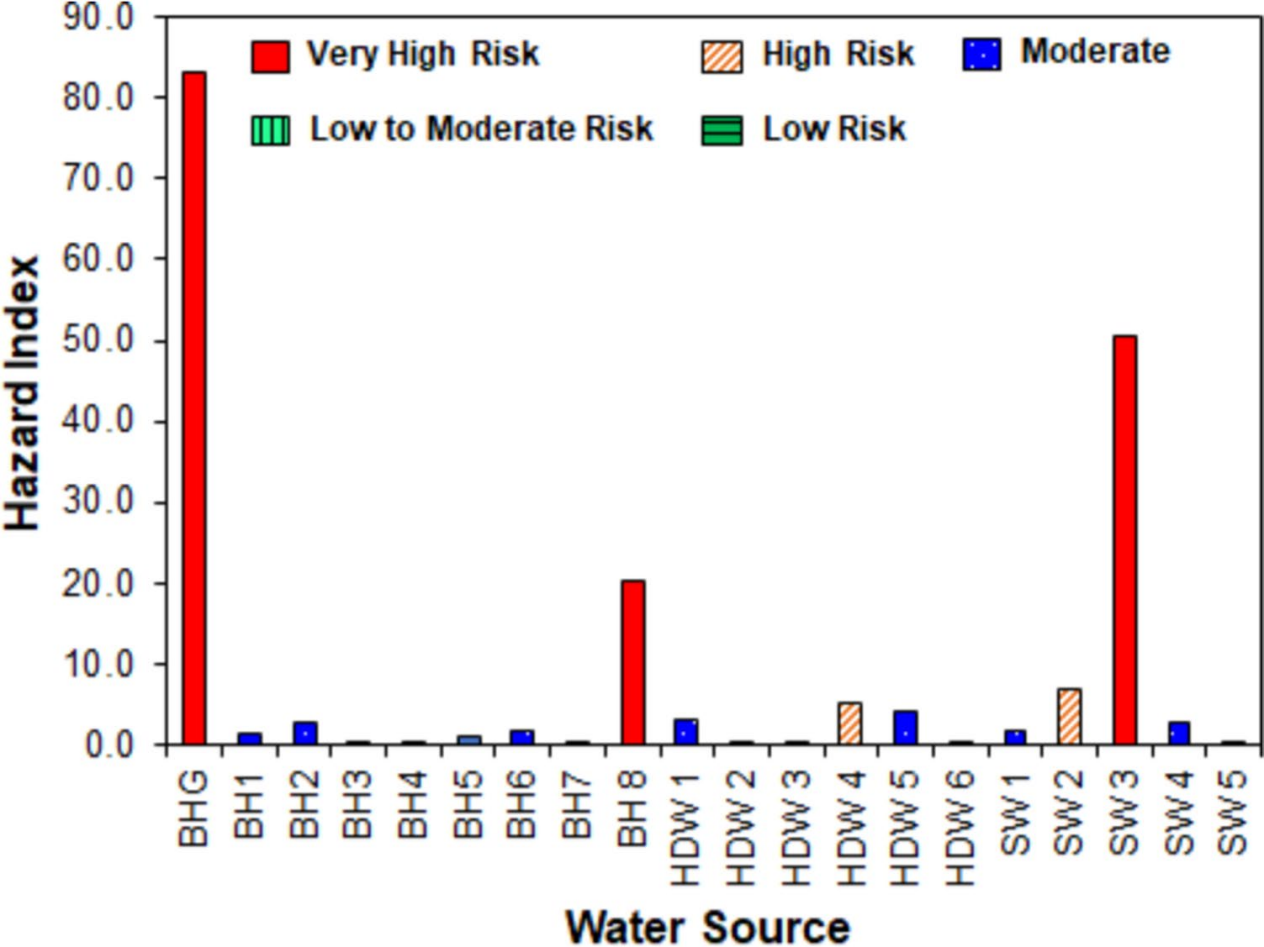
Hazard index of groundwater and surface water sources

Figure 9 presents the oral HI of the heavy metals and CN in the water sources (see Fig. S1 in the Supplementary Materials for a clearer presentation). Also, detailed results are presented in Table S10. The results showed that for all the water sources, 15.0% were evaluated to have a very high non-carcinogenic harmful health risk (HI > 10.0), 10.0% had a high risk (5.0 ≤ HI < 10.0), 40.0% had a moderate risk (1.0 ≤ HI < 5.0), 30.0% had a low to moderate risk (0.1 ≤ HI < 1.0), and 5.0% had a low non-carcinogenic harmful health risk (HI < 0.1). Among the different types of water sources, approximately 13.0% of the groundwater exhibited a very high non-carcinogenic health risk, nearly 7.0% had a high risk, 44.0% presented a moderate risk, approximately 33.0% were low to moderate risk, and about 7.0% showed a low risk. For the streams, 20.0% were estimated to have very high and high non-carcinogenic harmful health risks, 40.0% had moderate risk, and 20.0% were low to moderate risk. Overall, HI > 1.0 for 65.0% of the water sources, indicating that non-cancer risks are likely to occur (Cao et al. 2015), except for BH3, BH4, BH7, HDW2, HDW3, HDW6, and SW5. Awomeso et al. (2017) obtained similar results in their study within ASGM areas in Ijeshaland, Southwestern Nigeria, in which HI adverse effects of trace elements for adults were higher in the groundwater (about 1.0 × 10^–2^–2.8 × 10^–1^) than in streams (about 1.0 × 10^–2^–4.0 × 10^–2^). However, their HI values were less than 1.0, whereas extremely high values were found in this present study ranging from 0.2 to 83.2 for groundwater and 0.1–50.4 for surface water. The difference could be due to the distance between the water sources and the mining areas, which was not considered by Awomeso et al. (2017). Another reason is that land and soil characteristics (for instance texture and permeability) influence pollutant transfer mechanisms such as erosion (in surface water sources) and leaching and permeation (into groundwater) (Amano et al. 2020; Nguyen et al. 2011).

Mestanza‑Ramón et al. (2023) studied HI resulting from exposure to Hg in rivers in the Ecuadorian Amazon. They found that HI values of all the rivers used for recreational settings were lower than the threshold of acceptable risk (HI = 1.2 × 10^–2^ to 1.1 × 10^–1^ for adults and 1.8 × 10^–2^ to 1.5 × 10^–1^ for children). However, in 20 locations, the HI values of rivers for residential use (HI = 2.9 × 10^–1^ to 2.6) exceeded the safe exposure limit for children based on water ingestion (HI = 1). The HI value of SW5 was within the range of values for adults, while those for SW1 to SW4 were remarkably higher than values for adults found for river streams by Mestanza‑Ramón et al. (2023). Although non-carcinogenic, Hg’s persistent and bioaccumulative nature presents serious threats to the environment and human health, even at minimal concentrations (Feng et al. 2022).

#### Carcinogenic health risk

**Fig. 10:**
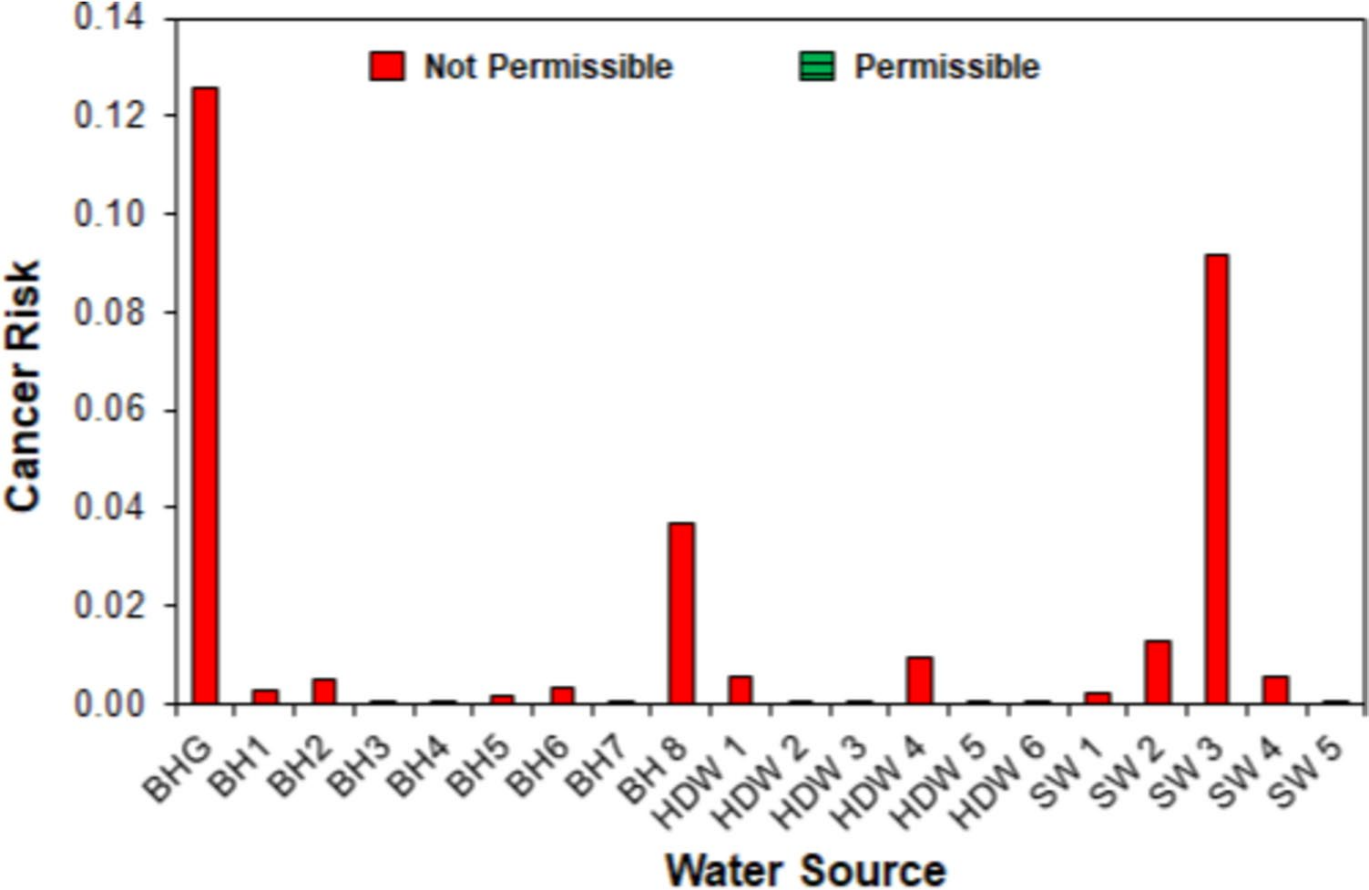
Cancer risk assessment of groundwater and surface water sources

Lifetime carcinogenic health risk was evaluated using only the CDI and CPSo of Cd, as the other heavy metals and CN do not contribute to cancer. A CR less than 1.0 × 10^−6^ is considered inconsequential, and the cancer risk can be ignored. Conversely, a CR up to or above 1.0 × 10^−4^ is considered harmful, indicating a bothersome cancer risk (Cao et al. 2015; USEPA 2005). The results in Fig. 10 show that 90.0% of all the water sources pose a carcinogenic risk when consumed, as the CR values of Cd are up to or higher than 1.0 × 10^−4^. By considering the nature of the water sources, around 87.0% of the groundwater and all the streams (100.0%) were considered harmful and have a very high potential to cause cancer over a lifetime. CR of 10^−4^ shows a possibility that 1 in 10,000 individuals could develop cancer (EPA 1986). Hence, from the results, 1 in 10 adults could develop cancer when they consume from BHG, and 1 in 100 adults could get cancer when they consume from BH8, SW2, or SW3. Also, 1 in 1000 adults are likely to develop cancer when they consume BH1, BH2, BH6, HDW1, HD4, SW1, or SW4, whereas 1 in 10,000 adults have the potential to develop cancer when they consume BH3, BH4, HDW3, HDW5, HDW6, or SW5. The results reveal that ASGM poses a high cancer risk to the community via the cumulative ingestion of contaminants in drinking water sources. However, children are more vulnerable as they accumulate more of the toxicant, even though they consume less water (Awomeso et al. 2017). Hence, they are more likely to develop cancer when they consume water sources within the ASGM area. Detailed results are presented in Table S10 and Fig. S2 in the Supplementary Material.

Cancer health risks for ASGM workers exposed to Cd and As in rivers in the Prestea Huni Valley District of Ghana were found to be above the acceptable range of 1.0 × 10^−6^ to 1.0 × 10^−4^, while the cancer risks for most of the sediments were within the acceptable range (Obiri et al. 2016). However, their study focused only on mine workers; hence, it was not extended to the water sources used by the communities and did not examine groundwater pollution. Total cancer risk between 7.0 × 10^–6^ and 2.5 × 10^–5^ has been found in stream waters within gold mining areas in the Ecuadorian Amazon (Galarza et al. 2023), and 1.4 × 10^–7^–1.9 × 10^–3^ in groundwater in Kerala, India (Krishna and Achari 2024). Lifetime cancer risks from Cd ingestion, ranging from about 8.5 × 10^–2^ to 1.1 × 10^–1^, have been reported for groundwater and table water sold in the Tudun Murtala area of Kano State, Nigeria (Salihu et al. 2019). Additionally, cancer risks from cumulative ingestion and dermal contact with Cr in drinking water have been reported in Khorramabad, Iran (Mohammadi et al. 2019) and Kolleru Lake in Andhra Pradesh, India (Sharma 2020). However, the water sources investigated in these studies were not within gold mining areas; hence, direct comparisons cannot be made. It must be emphasised that research assessing the carcinogenic health risk of drinking water sources within gold mining areas is limited in the literature.

This study provides valuable information for evaluating pollutants in streams, rivers, and groundwater sources within ASGM areas, along with their associated human health risks. These findings, therefore, offer scientific evidence that can compel regulatory bodies to establish environmental policies aimed at mitigating ecological pollution and the health risks resulting from ASGM activities.

Table 6 summarises the range of concentrations of the water quality parameters, physicochemical, heavy metals, as well as the range of WQI, HPI, and CR values of ground and surface water sources in the Asankrangwa district in Ghana and other gold mining regions in the world. These reported results have been discussed in detail in the previous sections.

**Table 6:** Comparison of physicochemical and heavy metals concentrations in water sources of this study (Asankrangwa district) with selected other locations in the world

Location	Turbidity (NTU)	EC (µS/cm)	TDS (mg/L)	TH (mg/L)	Alkalinity (mg/L)	CN (mg/L)	Hg (mg/L)	Cd (mg/L)	Fe	WQI	HPI	CR	References
Asankrangwa, Ghana (groundwater)	1.5–100.0	125.8–493.8	71.3–257.9	485.0–4948.0	14.5–105.0	0.0–0.3	4.0 × 10^–4^–7.2 × 10^–1^	3.0 × 10^–2^–5.9 × 10^–2^	1.3 × 10^–2^–8.9 × 10^–2^	39.7–395.1	163.1–1571.9	7.0 × 10^–5^–1.3 × 10^–1^	This study
% Exceeding permissible limits	53.3	0.0	0.0	93.3	0.0	6.7	80.0	100.0		20.0 ^a^	40.0 ^b^	87.0 ^c^	
Asankrangwa, Ghana (Surface water)	2.9–52.4	131.3–387.4	77.5–187.8	505.0–2804.0	77.5–187.8	0.0–1.2 × 10^–2^	1.0 × 10^–3^ –7.2 × 10^–2^	1.4 × 10^–2^–6.9 × 10^–2^	4.3 × 10^–2^–9.2 × 10^–2^	54.6–348.1	376.6–1119.8	1.7 × 10^–4^–9.2 × 10^–2^	This study
% Exceeding permissible limits	80.0	0.0	0.0	100.00	0.0	0.0	80.0	100.0		20.0 ^a^	60.0 ^b^	100.0 ^c^	
Rivers (Pra, Offin, and Birim), Ghana	–	49.3–1104.0	31.0–684.0	–	–	–	5.0 × 10^–6^–7.6 × 10^–3^	1.0 × 10^–6^–1.4 × 10^–3^	4.3 × 10^–1^	–	–	–	Bessah et al. (2021)
Bibiani area, Ghana (groundwater)	–	–	–	–	350.0	2.0 × 10^–3^–4.0 × 10^–2^	–	–	3.0	–	–	–	Gyawu-Asante et al. (2017)
Bibiani, Ghana (surface water)	–	–	–	–	350.0	8.0 × 10^–3^–2.0 × 10^–2^	–	–	1.0 × 10^–2^	–	–	–	Gyawu-Asante et al. (2017)
Newmont area, Ghana (surface water)	–	–	–	–	–	1.0 × 10^–2^–5.0 × 10^–2^	–	–	1.0 × 10^–2^	–	–	–	Asamoah-Boateng (2009)
Nangodi and Tinga rivers, Northern Ghana	–	–	–	–	–	–	3.8 × 10^–2^–6.4 × 10^–2^	5.3 × 10^–1^–2.3 × 10^–2^	–	–	–	–	Cobbina et al. (2015)
River Samre, Western Region, Ghana	–	–	–	–	–	–	1.0 × 10^–2^–2.0 × 10^–2^	2.0 × 10^–3^–1.1 × 10^–2^	–	–	–	–	Nkoom et al. (2013)
La Toma, Columbia (surface and drinking water)	1.8–8.7	4.6 × 10^–2^–7.6 × 10^–2^	–	–	–	–	1.7 × 10^–4^–9.7 × 10^–4^	–	–	–	–	–	Casso-Hartmann et al. (2022)
Ecuadorian Amazon (stream waters)	–	–	–	–	–	–	1.0 × 10^–3^–3.0 × 10^–3^	–	–	–	–	–	Mestanza‑Ramón et al. (2023)
Ecuadorian Amazon (stream waters)	–	–	–	–	–	–	–	0.2–0.8	227.0–558.0			7.0 × 10^–6^–2.5 × 10^–5^	Galarza et al. (2023)
Kerala, India (groundwater)	0.0–70.0	240.0–8100.0	50.0–1890.0	35.0–539.0	51.0–299.0	–	–	BDL	8.8 × 10^–2^–1.4	23.6–218.2	–	1.4 × 10^–7^–1.9 × 10^–3^	Krishna and Achari (2024)
Igade_Meshegu, Nigeria (groundwater)	–	342.0–610.0	–	–	–	–	1.0 × 10^–3^–0.1	0. 0–5.0 × 10^–3^	1.0 × 10^–3^–10.1	–	–	–	Sani et al. (2023)
Igade, Meshegu, Nigeria (river)	–	–	–	–	–	–	1.0 × 10^–3^–1.1	1.0 × 10^–3^–1.2 × 10^–2^	1.0 × 10^–3^–10.0	–	–	–	Sani et al. (2023)
Ogun State, Nigeria (surface water)	35.5–222.9	150.0–225.0	89.0–158.0	7.5–182.9	–	–	0.1–2.0	–	–	–	–	–	Olalekan et al. (2023)
Minna, Nigeria (River Kpapi)	–	67.0–186.0	42.9–119.0	12.0–100.0	–	–	–	2.0 × 10^–2^–6.0 × 10^–2^	–	2045.4–3087.5	–	–	Dukiya et al. (2023)
WHO/GSA limits	5.0	1000.0	1000.0	500.0	500.0	0.2 ^d^	1.0 × 10^–3^	3.0 × 10^–3^	0.3	–	–	1.0 × 10^–6 d^	GSA (2013); WHO (2011); ^d^ USEPA (2020)

^a^Water sources are unsuitable for consumption

^b^Water sources are highly contaminated

^c^Harmful with a very high potential to cause cancer over a lifetime

### Impact of artisanal and small-scale mining on human health

As explained in the ‘Chemical characteristics of water quality’ section, the borehole at the mining site (BHG) used by the miners contained an elevated concentration of CN (Table 2), which was far higher than the MCL of 0.20 mg/L (USEPA 2022). Consuming water containing CN above the MCL can cause adverse health problems such as damage to the nervous system, other neurological effects, tremors, and thyroid problems (USEPA 2022). The results in Table 4 reveal that 75.0% of all the water sources in the study area contained Hg concentrations higher than the GSA standard limit of 1.0 × 10^–3^ mg/L (GSA 2013). Hg causes kidney and renal problems, tremors, mental disturbances, gingivitis, neurological effects, and damage to the gastrointestinal tract and respiratory system (USEPA 2022). Also, Cd is carcinogenic and causes kidney damage (USEPA 2022; WHO 2011). The results revealed that 90.0% of all water sources posed carcinogenic health risks due to high levels of cadmium (Cd). High concentrations of Hg and Cd may adversely affect the endocrine system of individuals who drink the untreated, contaminated water sources (Nkoom et al. 2013).

Health statistics in the Amenfi West District revealed that 1.4% of the population in the Asankrangwa area (i.e. the district) suffers from some form of disability (Ghana Statistical Service 2014). Of that 1.4% population, 37.0% had sight-related illnesses, 32.8% had physical disabilities, and 14.9% suffered from emotional problems. The latter could be linked to the consumption of water sources containing high concentrations of Hg. The increase in cases of kidney diseases has been a matter of serious concern in Ghana, and it has been reported that Hg used by ASGM activities is a major contributory factor (Kusi-Ampofo and Boachie-Yiadom 2012). Previous investigations in gold mining areas in Ghana disclosed that diseases aggravated by mining include upper respiratory tract infections, Buruli ulcers, skin diseases, diarrhoea, fever, colds, and catarrh (Akabzaa and Darimani 2001; Duker et al. 2006; Yeboah 2008). Respiratory infections represented 27.0% of the reported diseases in some areas, followed by skin diseases (17.7%), while fever, diarrhoea, and other illnesses accounted for 13.6% (Yeboah 2008).

A study assessing the occupational exposure of 343 ASGM miners to Hg in Prestea, a gold mining town in Ghana, found that 46.7% of the miners had Hg in their urine (5.0–50.5 µg/L) above the recommended exposure limit of < 5.0 µg/L (Mensah et al. 2016). Similarly, a study involving 50 ASGM workers from Anhwiaso in the Ankobra River basin in Ghana found high concentrations of Hg in their blood (102.0 µg/L) and urine (34.5 µg/L) (Aidoo 2022). Similar findings have been reported in South Africa, Venezuela, and Brazil, where very high urine Hg levels of 50.0%, 48.3%, and 52.0%, respectively, above the recommended occupational exposure limits were found among ASG miners (Oosthuizen et al. 2010; Rojas et al. 2001; Silbergeld et al. 2002).

A retrospective study was conducted to analyse 20 years of autopsy records of the two topmost teaching hospitals in Ghana: Korle-Bu Teaching Hospital (KBTH) in Accra and Komfo Anokye Teaching Hospital (KATH) in Kumasi, from January 1994 to December 2013. The study found that 5.9% out of 94,309 deaths were caused by renal diseases and reported a rise in the renal disease mortality rate during the last few years (Adjei et al. 2019). A very recent report shows that kidney patients at KATH have quadrupled in the last decade, partly caused by ASGM activities. Hospital admissions have also increased threefold, while people seen as outpatients are four times greater than a decade ago. It has been further reported that the average age of persons in Ghana living with kidney diseases has now reduced to 40 years, which is a disturbing reduction from the normal 60 years from 10 years ago (Quaicoe 2022). The health reports indicate that ASGM activities lead to harmful health effects, which require critical attention.

## Conclusions

The quality of water sources used for consumption within artisanal and small-scale gold mining communities has been successfully evaluated by analysing the physicochemical water parameters, CN, and heavy metal concentrations. According to the water quality index, 40% of the water sources within the ASGM communities are unsuitable for consumption. A parameter of particular concern is turbidity, which was found in 20.0% of the water sources within 4.65 km of the mining sites and greatly exceeded the WHO limit of 5 NTU. This turbidity is caused by the discharge and permeation of particulates into the water sources. The study revealed that ASGM activities do not generally contribute to CN pollution of water sources in the nearby communities.

Heavy metal pollution indices indicated that 45.0% of the water sources are highly polluted, while 25.0% are moderately polluted; mainly influenced by the extremely high concentrations of Cd and Hg. The streams are more contaminated than the groundwaters, with contamination of streams caused by erosion and sedimentation from the mining sites. In contrast, groundwater pollution stems from the permeation of mining contaminants.

Health risk assessments indicated that 90.0% of the water sources within the ASGM communities are considered harmful and have a very high potential to cause cancer when consumed via oral means.

The findings of this study revealed that although heavy metal contamination is reduced and the water quality improved 5 km away from the mining sites, the exceedingly high Hg and Cd levels in the water sources within 30 km of ASGM sites are alarming. Therefore, critical measures are required from government authorities to ensure that efficient protocols are followed to prevent the discharge of mining waste into the environment. Efficient treatment of water sources is recommended to reduce pollutant concentrations below the threshold limits, rendering them wholesome for consumption.

## Supplementary Information

Below is the link to the electronic supplementary material.

Supplementary file1 (DOCX 70.8 KB)

## Data Availability

All the data associated with this study are available in this published article and its Supplementary Data files.

## Declarations

### Clinical trial number

This is not applicable.
