# Supplementary material for: Pollution and health risk assessment of drinking water sources within artisanal and small-scale gold mining areas: a case of Asankrangwa District in Ghana
Source: Environ Sci Pollut Res Int. 2026 Feb 10;33(8):3310–38. doi: 10.1007/s11356-026-37463-y (PMC13005872; doi:10.1007/s11356-026-37463-y)
Supplement: Supplementary file 1 — Supplementary file1 (DOCX 70.8 KB) [file 11356_2026_37463_MOESM1_ESM.docx]

**Pollution and Health Risk Assessment of Drinking Water Sources within Artisanal and Small-Scale Gold Mining Areas: The Case of Asankrangwa District in Ghana**

Eric Danso-Boateng^1, *^_,_ Ebenezer Adom^2^, Prince Appiah Owusu^3^ and Roland Songotu Kabange^3^

^1^School of Chemical and Process Engineering, University of Leeds, Leeds, LS2 9JT, UK

^2^Department of Chemical Engineering, Kwame Nkrumah University of Science and Technology, Private Mail Bag, University Post Office, Kumasi, Ghana.

^3^Department of Civil Engineering, Kumasi Technical University, P.O. Box 854 Kumasi, Ghana.

*Email: Correspondence: e.danso-boateng@leeds.ac.uk

ORCID ID: 0000-0003-3555-3118

**Table S1.** Summary of geographical location of study site

| **Water Source** | **Sample ID** | **Location** | **Name of Area** | **Distance to Focal Point (km)** |
| --- | --- | --- | --- | --- |
| Bore hole | BHG | 5^o^49’35’11’’N 2^o^25’36”74W | Galamsey site | 0.00 |
| Borehole 1 | BH1 | 5^o^48’1.57” N 2^o^25’8.31” W | Education | 2.97 |
| Borehole 2 | BH2 | 5^o^48’26.4” N 2^o^26’9.6” W | Kwasibokro 1 | 30.11 |
| Borehole 3 | BH3 | 5^o^48’20.4” N 2^o^26’9.3” W | Royal | 2.40 |
| Borehole 4 | BH4 | 5^o^48’21.1” N 2^o^26’8.7” W | Kwasibokro 1b | 2.46 |
| Borehole 5 | BH5 | 5^o^48’51” N 2^o^26’3.4” W | Kwasibokro 2b | 1.56 |
| Borehole 6 | BH6 | 5^o^48’22.2” N2^o^25’31.8” W | Malta, revenue | 2.19 |
| Borehole 7 | BH7 | 5^o^48’53.2” N 2^o^25’45.1” W | Education | 1.30 |
| Borehole 8 | BH8 | 5^o^48’11” N 2^o^26’4.1” W | Nursing Training | 2.68 |
| Hand dug 1 | HDW1 | 5^o^48’25” N 2^o^26’48” W | Blockso | 3.01 |
| Hand dug 2 | HDW2 | 5^o^48’28.9” N 2^o^26’40.1” W | Kwasibokro 2 | 2.78 |
| Hand dug 3 | HDW3 | 5^o^48.4’77” N 2^o^26.1’21” W | Last stop | 2.83 |
| Hand dug 4 | HDW4 | 5^o^46.2’94” N 2^o^25’51” W | Gyarman | 6.52 |
| Hand dug 5 | HDW5 | 5^o^48’26” N 2^o^26’48” W | Malta | 3.05 |
| Hand dug 6 | HDW6 | 5^o^48’52” N 2^o^25’48.5” W | Nkwanta Twam | 4.18 |
| Stream 1 | SW1 | 5^o^47.5’75” N 2^o^25.6’98” W | Salami Uptown | 4.65 |
| Stream 2 | SW2 | 5^o^47’52.6” N 2^o^25’34.9” W | Blockso newtown | 3.11 |
| Stream 3 | SW3 | 5^o^48.7’36” N 2^o^26.7’45” W | Amoman | 2.81 |
| Stream 4 | SW4 | 5^o^47.1’80” N 2^o^28.2’58” W | Adowaho | 6.44 |
| Stream 5 | SW5 | 5^o^49.2’25” N 2^o^26.4’81” W | Nakaba | 1.32 |
| BH = Borehole; BHG = Borehole at mining site; HDW = Hand-dug well; SW = Surface water. | | | | |

**Table S2.** Method detection limit and quantitation limit in mgL^−1^ for the selected metals

| **Metals** | **MDL** | **LOQ** | **IDL** | **% Recovery** | **% RSD** |
| --- | --- | --- | --- | --- | --- |
| Pb | 0.082 | 1.623 | 0.08 | 96.0–107.0 | 0.03– 4.9 |
| Hg | 0.051 | 0.179 | 0.04 | 90.8–104.2 | 0.04–2.9 |
| Mn | 0.02 | 0.294 | 0.01 | 92.8–103.5 | 0.03–3.7 |
| Cu | 0.009 | 0.231 | 0.005 | 91.5–100.5 | 0.2–7.7 |
| Cd | 0.054 | 0.115 | 0.01 | 96–101.6 | 0.03–3.1 |
| Fe | 0.03 | 0.107 | 0.06 | 91.7–100.4 | 0.03–3.7 |

**Table S.3** Relative weight parameters for calculating WQI

| **Parameter** | **WHO/GSA standard** | **Weight (*w_i_*)** | **Relative weight (*W_i_*)** |
| --- | --- | --- | --- |
| pH | 6.5-8.5 | 4 | 0.111 |
| Turbidity | 5.0 NTU | 5 | 0.139 |
| Electrical conductivity | 1000.0 mg/L | 4 | 0.111 |
| Total dissolved solids | 1000.0 mg/L | 5 | 0.139 |
| Alkalinity | 300.0 mg/L | 4 | 0.111 |
| Total hardness | 500.0 mg/L | 2 | 0.056 |
| Ca^2+^ | 75.0 mg/L | 2 | 0.056 |
| Mg^2+^ | 50.0 mg/L | 2 | 0.056 |
| Cl^-^ | 250.0 mg/L | 3 | 0.083 |
| CN | 0.07 mg/L | 5 | 0.139 |
|  |  | ∑*w_i_* = 36.0 | ∑*W_i_* = 1.0 |

**Table S.4** HPI calculation for each parameter

| **Heavy metals** | **Standard acceptable**  **value (*S_i_*) (ppb)** | **Highest acceptable**  **value (*I_i_*) (ppb)** | **Unit weightage**  **(*W_i_*)** |
| --- | --- | --- | --- |
| Fe | 300.0 | 100.0 | 0.003 |
| Cd | 10.0 | 3.0 | 0.100 |
| Cu | 2000.0 | 50.0 | 0.001 |
| Pb | 15.0 | 10.0 | 0.067 |
| Mn | 400.0 | 100.0 | 0.003 |
| Hg | 300.0 | 100.0 | 0.003 |

**Test for normality of water quality data**

Table S.5 presents the results of the Shapiro-Wilk normality test for 21 water quality parameters. The degrees of freedom (DF), test statistic, p-value, and decision at a 5% significance level are reported for each parameter. Elements with p ≤0.05 are considered to deviate significantly from normal distribution. The test results indicate that most parameters do not follow a normal distribution and therefore, it is necessary to employ non-parametric statistical techniques.

**Table S.5** Shapiro-Wilk normality test for 21 water quality parameters

| Parameters | DF | Statistics | p-value | Decision at level (5%) |
| --- | --- | --- | --- | --- |
| pH | 20 | 0.91395 | 0.07583 | Can't reject normality |
| Mg^2+^, mg/l | 20 | 0.76516 | 2.73 x 10^-4^ | Reject normality |
| Ca^2+^, mg/l | 20 | 0.88399 | 0.02089 | Reject normality |
| Total Hardness, mg/l | 20 | 0.87902 | 0.01699 | Reject normality |
| Alkalinity, mg/l | 20 | 0.86776 | 0.01073 | Reject normality |
| CN, mg/l | 20 | 0.34173 | <0.0001 | Reject normality |
| Turbidity, NTU | 20 | 0.6225 | <0.0001 | Reject normality |
| TDS, mg/l | 20 | 0.84947 | 0.00522 | Reject normality |
| Conductivity, (µS/cm) | 20 | 0.81734 | 0.00159 | Reject normality |
| Cl^-^ | 20 | 0.57087 | <0.0001 | Reject normality |
| HCO_3_ | 20 | 0.87284 | 0.01318 | Reject normality |
| SO_4_ | 20 | 0.78299 | 4.87E-04 | Reject normality |
| Na^+^ | 20 | 0.90311 | 0.04719 | Reject normality |
| K^+^ | 20 | 0.92003 | 0.09923 | Can't reject normality |
| Fe | 17 | 0.94207 | 0.3435 | Can't reject normality |
| Cd | 20 | 0.97318 | 0.82009 | Can't reject normality |
| Hg | 20 | 0.50845 | <0.0001 | Reject normality |
| Cu | -- | -- | -- | a* |
| Mn | 4 | 0.69751 | 0.01086 | Reject normality |
| Pb | -- | -- | -- | a* |

**Table S6.**  Evaluation of water quality based on WQI

| Sample ID | Distance from ASGM site (km) | WQI | WQI rating |
| --- | --- | --- | --- |
| BHG | 0.00 | 319.21 | Unsuitable |
| BH1 | 2.97 | 45.75 | Excellent |
| BH2 | 30.11^a^ | 39.69 | Excellent |
| BH3 | 2.4 | 395.14 | Unsuitable |
| BH4 | 2.46 | 50.16 | Good |
| BH5 | 15.6 | 66.95 | Good |
| BH6 | 2.19 | 127.79 | Poor |
| BH7 | 7.3 | 53.09 | Good |
| BH8 | 12.68 | 45.20 | Excellent |
| HDW1 | 13.1 | 67.27 | Good |
| HDW2 | 2.78 | 251.36 | Very Poor |
| HDW3 | 2.83 | 349.87 | Unsuitable |
| HDW4 | 6.52 | 55.83 | Good |
| HDW5 | 13.5 | 72.71 | Good |
| HDW6 | 4.18 | 54.11 | Good |
| SW1 | 4.65 | 172.12 | Poor |
| SW2 | 3.11 | 348.06 | Unsuitable |
| SW3 | 2.81 | 260.94 | Very Poor |
| SW4 | 6.44 | 73.90 | Good |
| SW5 | 7.2 | 54.59 | Good |
| ^a^ Control point. BH = Borehole; BHG = Borehole at mining site; HDW = Hand-dug well; SW = Surface water. | | | |

**Table S7.**  Evaluation of water quality based on HPI and HEI

| Sample ID | Distance from  ASGM site (km) | HPI | Degree of  pollution | HEI | Degree of pollution |
| --- | --- | --- | --- | --- | --- |
| BHG | 0.00 | 1571.89 | High | 39.14 | High |
| BH1 | 2.97 | 1122.95 | High | 35.71 | High |
| BH2 | 30.11^a^ | 163.05 | Low | 14.83 | Low |
| BH3 | 2.40 | 1271.63 | High | 39.63 | High |
| BH4 | 2.46 | 497.09 | Medium | 32.91 | High |
| BH5 | 15.56 | 376.52 | Low | 8.83 | Low |
| BH6 | 2.19 | 1309.90 | High | 32.18 | High |
| BH7 | 7.30 | 432.05 | Medium | 23.11 | Medium |
| BH8 | 12.68 | 215.15 | Low | 7.31 | Low |
| HDW1 | 13.01 | 322.78 | Low | 7.51 | Low |
| HDW2 | 2.78 | 1240.04 | High | 32.71 | High |
| HDW3 | 2.83 | 1240.04 | High | 38.95 | High |
| HDW4 | 6.52 | 767.15 | Medium | 21.36 | Medium |
| HDW5 | 13.05 | 383.74 | Low | 10.71 | Low |
| HDW6 | 4.18 | 682.94 | Medium | 24.04 | Medium |
| SW1 | 4.65 | 1119.84 | High | 31.40 | High |
| SW2 | 3.11 | 1079.31 | High | 35.40 | High |
| SW3 | 12.81 | 1068.63 | High | 35.28 | High |
| SW4 | 6.44 | 376.55 | Low | 9.03 | Low |
| SW5 | 7.20 | 692.66 | Medium | 16.40 | Medium |
| ^a^ Control point. BH = Borehole; BHG = Borehole at mining site; HDW = Hand-dug well; SW = Surface water. | | | | | |

**Table S8.**  Chronic daily intake (CDI) of chemical substances in the water sources

| **Sample ID** | **Distance from**  **ASGM site (km)** | **Fe**  **(mg/kg-day)** | **Mn**  **(mg/kg-day)** | **Cd**  **(mg/kg-day)** | **Hg**  **(mg/kg-day)** | **CN**  **(mg/kg-day)** |
| --- | --- | --- | --- | --- | --- | --- |
| BHG | 0.00 | 1.37 x 10^-3^ | 6.14 x 10^-3^ | 1.14 x 10^-3^ | 2.06 x 10^-2^ | 8.63 x 10^-3^ |
| BH1 | 2.97 | – | – | 8.86 x 10^-4^ | 4.29 x 10^-4^ | 2.86E-05 |
| BH2 | 30.11^a^ | 7.14 x 10^-4^ | – | 1.09 x 10^-3^ | 7.71 x 10^-4^ | 1.14 x 10^-4^ |
| BH3 | 2.40 | 3.71 x 10^-4^ | – | 1.29 x 10^-3^ | 2.86 x 10^-5^ | 2.86 x 10^-5^ |
| BH4 | 2.46 | 1.40 x 10^-3^ | – | 1.51 x 10^-3^ | 8.57 x 10^-5^ | 8.57 x 10^-5^ |
| BH5 | 15.56 | 2.54 x 10^-3^ | – | 1.69 x 10^-3^ | 2.57 x 10^-4^ | 1.14 x 10^-4^ |
| BH6 | 2.19 | 2.26 x 10^-3^ | – | 1.20 x 10^-3^ | 5.43 x 10^-4^ | 0.00 |
| BH7 | 7.30 | 1.17 x 10^-3^ | – | 8.86 x 10^-4^ | 1.14 x 10^-5^ | 0.00 |
| BH8 | 12.68 | 2.03 x 10^-3^ | – | 1.23 x 10^-3^ | 6.09 x 10^-3^ | 0.00 |
| HDW1 | 13.01 | – | 1.43 x 10^-4^ | 1.46 x 10^-3^ | 9.14 x 10^-4^ | 0.00 |
| HDW2 | 2.78 | 3.14 x 10^-4^ | – | 8.57 x 10^-4^ | 1.14 x 10^-5^ | 2.00 x 10^-4^ |
| HDW3 | 2.83 | 1.20 x 10^-3^ | – | 1.40 x 10^-3^ | 2.86 x 10^-5^ | 1.43 x 10^-4^ |
| HDW4 | 6.52 | – | – | 1.17 x 10^-3^ | 1.57 x 10^-3^ | 8.57 x 10^-5^ |
| HDW5 | 13.05 | 1.20 x 10^-3^ | – | 1.46 x 10^-3^ | 5.71 x 10^-5^ | 2.31 x 10^-3^ |
| HDW6 | 4.18 | 1.49 x 10^-3^ | – | 1.20 x 10^-3^ | 1.14 x 10^-4^ | 1.14 x 10^-4^ |
| SW1 | 4.65 | 2.63 x 10^-3^ | 6.86 x 10^-4^ | 1.49 x 10^-3^ | 3.71 x 10^-4^ | 3.43 x 10^-4^ |
| SW2 | 3.11 | 2.49 x 10^-3^ | 2.57 x 10^-4^ | 1.00 x 10^-3^ | 2.06 x 10^-3^ | 2.86 x 10^-5^ |
| SW3 | 12.81 | 2.23 x 10^-3^ | – | 4.00 x 10^-4^ | 1.51 x 10^-2^ | 1.14 x 10^-4^ |
| SW4 | 6.44 | 1.71 x 10^-3^ | – | 1.51 x 10^-3^ | 8.86 x 10^-4^ | 0.00 |
| SW5 | 7.20 | 1.23 x 10^-3^ | – | 1.97 x 10^-3^ | 2.86 x 10^-5^ | 0.00 |
| ^a^ Control point. BH = Borehole; BHG = Borehole at mining site; HDW = Hand-dug well; SW = Surface water. | | | | | | |

**Table S9.**  Hazard quotient (HQ) of chemical substances in the water sources

| **Sample ID** | **Distance from**  **ASGM site (km)** | **Fe** | **Mn** | **Cd** | **Hg** | **CN** |
| --- | --- | --- | --- | --- | --- | --- |
| BHG | 0.00 | 1.96 x 10^-3^ | 4.39 x 10^-2^ | 2.29 x 10^-3^ | 68.76 | 14.38 |
| BH1 | 2.97 | 0.00 | 0.00 | 1.77 x 10^-3^ | 1.43 | 0.05 |
| BH2 | 30.11^a^ | 1.02 x 10^-3^ | 0.00 | 2.17 x 10^-3^ | 2.57 | 0.19 |
| BH3 | 2.40 | 5.31 x 10^-4^ | 0.00 | 2.57 x 10^-3^ | 0.10 | 0.05 |
| BH4 | 2.46 | 2.00 x 10^-3^ | 0.00 | 3.03 x 10^-3^ | 0.29 | 0.14 |
| BH5 | 15.56 | 3.63 x 10^-3^ | 0.00 | 3.37 x 10^-3^ | 0.86 | 0.19 |
| BH6 | 2.19 | 3.22 x 10^-3^ | 0.00 | 2.40 x 10^-3^ | 1.81 | 0.00 |
| BH7 | 7.30 | 1.67 x 10^-3^ | 0.00 | 1.77 x 10^-3^ | 0.04 | 0.00 |
| BH8 | 12.68 | 2.90 x 10^-3^ | 0.00 | 2.46 x 10^-3^ | 20.29 | 0.00 |
| HDW1 | 13.01 | 0.00 | 1.02 x 10^-3^ | 2.91 x 10^-3^ | 3.05 | 0.00 |
| HDW2 | 2.78 | 4.49 x 10^-4^ | 0.00 | 1.71 x 10^-3^ | 0.04 | 0.33 |
| HDW3 | 2.83 | 1.71 x 10^-3^ | 0.00 | 2.80 x 10^-3^ | 0.10 | 0.24 |
| HDW4 | 6.52 | 0.00 | 0.00 | 2.34 x 10^-3^ | 5.24 | 0.14 |
| HDW5 | 13.05 | 1.71 x 10^-3^ | 0.00 | 2.91 x 10^-3^ | 0.19 | 3.86 |
| HDW6 | 4.18 | 2.12 x 10^-3^ | 0.00 | 2.40 x 10^-3^ | 0.38 | 0.19 |
| SW1 | 4.65 | 3.76 x 10^-3^ | 4.90 x 10^-3^ | 2.97 x 10^-3^ | 1.24 | 0.57 |
| SW2 | 3.11 | 3.55 x 10^-3^ | 1.84 x 10^-3^ | 2.00 x 10^-3^ | 6.86 | 0.48 |
| SW3 | 12.81 | 3.18 x 10^-3^ | 0.00 | 8.00 x 10^-4^ | 50.19 | 0.19 |
| SW4 | 6.44 | 2.45 x 10^-3^ | 0.00 | 3.03 x 10^-3^ | 2.95 | 0.00 |
| SW5 | 7.20 | 1.76 x 10^-3^ | 0.00 | 3.94 x 10^-3^ | 0.10 | 0.00 |
| ^a^ Control point. BH = Borehole; BHG = Borehole at mining site; HDW = Hand-dug well;  SW = Surface water. | | | | | | |

**Table S10.**  Hazard index (HI) and cancer risk (CR) of the water sources

| **Sample ID** | **Distance from**  **ASGM site (km)** | **HI** | **HI Rating** | **CR** | **CR Rating** |
| --- | --- | --- | --- | --- | --- |
| BHG | 0.00 | 83.19 | Very High Risk | 1.26 x 10^-1^ | Not Permissible |
| BH1 | 2.97 | 1.48 | Moderate Risk | 2.61 x 10^-3^ | Not Permissible |
| BH2 | 30.11^a^ | 2.77 | Moderate Risk | 4.71 x 10^-3^ | Not permissible |
| BH3 | 2.40 | 0.15 | Low to Moderate Risk | 1.74 x 10^-4^ | Not Permissible |
| BH4 | 2.46 | 0.43 | Low to Moderate Risk | 5.23 x 10^-4^ | Not Permissible |
| BH5 | 15.56 | 1.05 | Moderate Risk | 1.57 x 10^-3^ | Not permissible |
| BH6 | 2.19 | 1.82 | Moderate Risk | 3.31 x 10^-3^ | Not permissible |
| BH7 | 7.30 | 0.04 | Low Risk | 6.97 x 10^-5^ | Permissible |
| BH8 | 12.68 | 20.29 | Very High Risk | 3.71 x 10^-2^ | Not permissible |
| HDW1 | 13.01 | 3.05 | Moderate Risk | 5.58 x 10^-3^ | Not permissible |
| HDW2 | 2.78 | 0.37 | Low to Moderate Risk | 6.97 x 10^-5^ | Permissible |
| HDW3 | 2.83 | 0.34 | Low to Moderate Risk | 1.74 x 10^-4^ | Not Permissible |
| HDW4 | 6.52 | 5.38 | High Risk | 9.59 x 10^-3^ | Not permissible |
| HDW5 | 13.05 | 4.05 | Moderate Risk | 3.49 x 10^-4^ | Not Permissible |
| HDW6 | 4.18 | 0.58 | Low to Moderate Risk | 6.97 x 10^-4^ | Not Permissible |
| SW1 | 4.65 | 1.82 | Moderate Risk | 2.27 x 10^-3^ | Not permissible |
| SW2 | 3.11 | 6.91 | High Risk | 1.25 x 10^-2^ | Not permissible |
| SW3 | 12.81 | 50.38 | Very High Risk | 9.18 x 10^-2^ | Not permissible |
| SW4 | 6.44 | 2.96 | Moderate Risk | 5.40 x 10^-3^ | Not permissible |
| SW5 | 7.20 | 0.10 | Low to Moderate Risk | 1.74 x 10^-4^ | Not Permissible |
| ^a^ Control point. BH = Borehole; BHG = Borehole at mining site; HDW = Hand-dug well;  SW = Surface water. | | | | | |


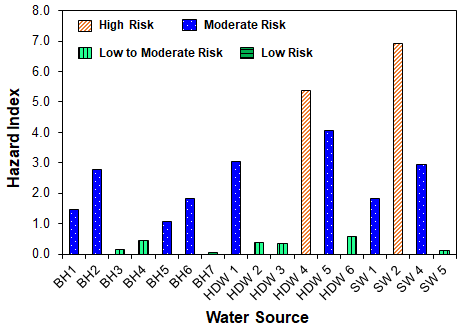


**Figure S1.** Hazard index of groundwater and surface water sources. Very high values for BHG, BH8, and SW3 have been removed for clarity.


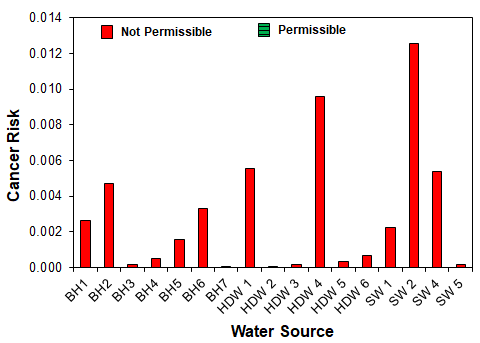


**Figure S2.** Cancer risk assessment of groundwater and surface water sources. Very high values for BHG, BH8 and SW3 were removed for clarity.
